# Mapping Global Research Trends on Aflatoxin M1 in Dairy Products: An Integrative Review of Prevalence, Toxicology, and Control Approaches

**DOI:** 10.3390/foods15010166

**Published:** 2026-01-03

**Authors:** Marybel Abi Rizk, Lea Nehme, Selma P. Snini, Hussein F. Hassan, Florence Mathieu, Youssef El Rayess

**Affiliations:** 1 Department of Agriculture and Food Engineering, School of Engineering, Holy Spirit University of Kaslik, Jounieh P.O. Box 446, Lebanon; marybelabirizk@usek.edu.lb (M.A.R.); leanehme@usek.edu.lb (L.N.); 2 Processing and Biosystems (ProBio) Multidisciplinary Research Group, Holy Spirit University of Kaslik, Jounieh P.O. Box 446, Lebanon; 3 Laboratoire de Génie Chimique, Université de Toulouse, CNRS, INPT, UPS, 31326 Toulouse, France; selma.snini@toulouse-inp.fr (S.P.S.); florence.mathieu@toulouse-inp.fr (F.M.); 4 Department of Nutrition and Food Science, School of Arts and Sciences, Lebanese American University, Beirut 1102-2801, Lebanon; hussein.hassan@lau.edu.lb

**Keywords:** AFM_1_ carry-over, milk safety, exposure assessment, bibliometric analysis, mitigation strategies, regulatory standards, mycotoxin contamination

## Abstract

Aflatoxin M_1_ (AFM_1_), a hydroxylated metabolite of aflatoxin B_1_ (AFB_1_), is a potent hepatotoxic and carcinogenic compound frequently detected in milk and dairy products. Its thermal stability and resistance to processing make it a persistent public health concern, especially in regions prone to fungal contamination of animal feed. This review integrates bibliometric mapping (2015–2025) with toxicological and mitigation perspectives to provide a comprehensive understanding of AFM_1_. The bibliometric analysis reveals a sharp global rise in research output over the last decade, with Iran, China, and Brazil emerging as leading contributors and Food Control identified as the most prolific journal. Five research clusters were distinguished: feed contamination pathways, analytical detection, toxicological risk, regulatory frameworks, and mitigation strategies. Toxicological evidence highlights AFM_1_’s mutagenic and hepatocarcinogenic effects, intensified by co-exposure to other mycotoxins or hepatitis B infection. Although regulatory limits range from 0.025 µg/kg in infant formula (EU) to 0.5 µg/kg in milk (FDA), non-compliance remains prevalent in developing regions. Current mitigation approaches—adsorbents (bentonite, zeolite), oxidation (ozone, hydrogen peroxide), and biological detoxification via lactic acid bacteria and yeasts—show promise but require optimization for industrial application. Persistent challenges include climatic variability, inadequate feed monitoring, and heterogeneous regulations. This review emphasizes the need for harmonized surveillance, improved analytical capacity, and sustainable intervention strategies to ensure dairy safety and protect consumer health.

## 1. Introduction

The World Health Organization (WHO) has recognized aflatoxins, particularly aflatoxin B_1_, as a contributing factor in human liver cancer [1]. In 2022, liver cancer accounted for over 758,700 deaths worldwide [2]. According to global burden estimates, 25,200 to 155,000 liver cancer cases are attributed annually to aflatoxin B_1_ exposure, especially in regions with high maize consumption and endemic hepatitis B infection [3]. Although chronic low-dose exposure to aflatoxins poses long-term risks, acute aflatoxicosis outbreaks have been reported in many fatality cases, with rates as high as 76%, affecting vulnerable populations in low- and middle-income countries [4]. Aflatoxins have a notable economic implication, including reduced livestock productivity, increased veterinary and health care costs, and contaminated product recall or rejection [5]. Moreover, there is a synergistic effect between different mycotoxins or between mycotoxins and other contaminants, such as Microcystin-LR, which can amplify toxicity [6]. That is why national and international authorities have developed regulations to set an aflatoxin limit in milk and feed [7,8].

Aflatoxin contamination extends beyond public health concerns, as it affects global trade, agricultural sustainability, and national economies. In the United States, due to aflatoxin contamination, there are U.S. corn losses between USD 52 million and USD 1.68 billion annually, especially during the drought season [9]. Moreover, in the sub-Saharan region, the impact is more significant due to limited testing and control. In Kenya, Senerwa et al. [10] estimated that the dairy industry loses over USD 172 million annually due to aflatoxin contamination of feed and milk. The figures illustrate how aflatoxin contamination affects the entire dairy value chain, starting from feed production and extending to final dairy products. Moreover, strict regulatory limits, especially in developed countries, lead to rejected exports, reducing foreign exchange revenues and limiting growth in agri-food sectors of many developing countries.

Different classes of mycotoxins are identified based on variation in their chemical structure and toxicological effects. Among these mycotoxins, one of the most well-known groups is aflatoxin, which is produced by certain *Aspergillus* species, such as *Aspergillus flavus*, *Aspergillus parasiticus*, and *Aspergillus nomius* [11]. Aflatoxin B_1_ (AFB_1_), Aflatoxin B_2_, Aflatoxin G_1_, Aflatoxin G_2_, and Aflatoxin M_1_ have been the most studied among the 20 different identified aflatoxins [1]. The International Agency for Research on Cancer (IARC) has classified aflatoxin B_1_ (AFB_1_), which has the highest level of toxicity, as a Group 1 carcinogen. Aflatoxin M_1_ (AFM_1_), a hydroxylated metabolite of aflatoxin B_1_ found in milk and dairy products, was originally classified as a Group 2B carcinogen, previously considered possibly carcinogenic to humans. However, in 2002, it was reclassified as Group 1 due to its confirmed association with hepatocellular carcinoma (HCC) and its mutagenicity and genotoxic effect, confirming its status as a known human carcinogen [12]. Recent studies continue to report the occurrence of AFM_1_ in milk and its associated risks, emphasizing its ongoing relevance as a food safety concern [13,14,15].

Aflatoxin biosynthesis pathway consists of at least 27 enzymatic steps [16] and around 25 to 30 genes [17] encoded within a gene cluster in the fungal genome. These genes govern the transformation of acetate units via the polyketide pathway into aflatoxin precursors, which are further enzymatically altered to yield the final toxic compounds [18]. The assigned letters “G” and “B” refer to the detected fluorescent color seen under exposure to ultraviolet light, while “M” refers to AFB_1_’s secondary metabolite, which finds its way in milk and dairy products [19].

Aflatoxin B_1_ is known to contaminate a wide range of food and feed and is not limited to any region [20]. Escola et al. [7] showed that 60 to 80% of worldwide crops are contaminated by mycotoxins. In fact, aflatoxins are ubiquitous and can be present in various crops, cereals, maize, nuts, and others. Mahato et al. [21] showed that aflatoxins were found in 36.7% of tested cereal crops, corn, rice, barley, wheat, and sorghum, which are major components of the global diet. As a consequence, animal health is severely impacted by the consumption of contaminated animal feed. In Pakistan, an aflatoxicosis outbreak in a dairy herd resulted in a 75% abortion rate and an 18.8% decrease in milk yield [22]. Once consumed, aflatoxin B_1_ is metabolized in the liver of dairy animals and excreted in the milk as AFM_1_. Due to its stability, AFM_1_ can resist many heat treatments, posing a significant public concern. A meta-analysis revealed that 53% of lactating women’s breast milk samples were contaminated with AFM_1_, with the highest percentage observed in Eastern Africa [23]. In fact, tropical and subtropical regions are considered the most affected regions for aflatoxin contamination due to their warm and humid climates. These environmental factors combined with inadequate storage conditions and limited regulations, create an ideal state for the growth of aflatoxin-producing fungi [24].

Climate change will further exacerbate this issue as rising temperatures and drought events are expected to elevate the fungi producing aflatoxin B_1_ levels in dairy cattle feed, thereby increasing the occurrence of aflatoxin M_1_ in milk and dairy products [25]. Modeling projects indicate that by 2031–2040, over 89% of U.S. Corn Belt counties may experience higher aflatoxin due to climate warming [26]. Other modeling studies suggest that an increase of 2 °C would elevate aflatoxin contamination, making it a serious food safety risk, even in currently low-risk regions such as Southern Europe [27]. Therefore, aflatoxin is considered a dynamic and evolving food safety issue requiring monitoring strategies and multidisciplinary mitigation techniques.

Although extensive research has been conducted on AFM_1_ in dairy products, covering its occurrence, toxicity, and mitigation approaches, there is still a lack of comprehensive reviews that bring these dimensions together. In particular, no study has integrated these aspects with a bibliometric and scientometric analysis to provide a cohesive picture of research progress. This review addresses this gap by offering an integrated analysis of AFM_1_ in dairy over the past decade, combining global research trends, prevalence data, regulatory frameworks, health impacts, mitigation strategies, and processing effects. By contextualizing existing knowledge, this study not only synthesizes what is known but also identifies critical gaps and future research priorities needed to enhance dairy safety worldwide.

## 2. Bibliometric and Scientometric Analysis of Aflatoxin M_1_ Research in Dairy

A bibliometric study provides a quantitative approach to research development based on statistical and mathematical methods. It helps to evaluate and regroup the scientific literature available, providing a valuable insight into the evolution and the impact of a certain research area. Bibliometric analysis was performed previously on several topics related to health [28] and food [29,30,31]. Therefore, it is necessary to evaluate the literature available and spot the research gaps in this area. Data were collected in February 2025 from the Scopus database. Scopus was selected because it offers a broad multidisciplinary coverage among citation databases and includes a wide range of food science and mycotoxin journals. In addition, it provides a built-in tool for metadata extraction suitable for bibliometric mapping. The exported database included citation information such as authors, author IDs, article titles, publication year, journal source title, volume, issue, article number, page range, page count, number of citations, DOI, direct links, author affiliations, presence of abstract, author and index keywords, correspondence address, publisher, document type, publication stage, open-access status, source database, and EID.

### 2.1. Research Strategy

An initial search was conducted in the Scopus database using the following keywords: Aflatoxin M_1_, Milk, Milk Products, Dairy Product, Cheese, Milk Powder, and Yogurt across the article title, abstract, and keyword fields. The literature search covered publications from January 2015 to February 2025. This search yielded a total of 960 records. After screening for relevance based on title and abstract, and removing duplicates, editorials, English-based articles, and articles not directly related to AFM_1_ in milk or dairy products, 804 articles were selected for inclusion in the final analysis. The dataset was exported with full metadata, including authorship, article title, publication year, journal source, volume, citations, digital object identifiers (DOI), affiliations, abstracts, author and index keywords, correspondence information, publisher, and document type. The dataset was processed and analyzed using the Bibliometrix R-package (4.3.3) through the Biblioshiny interface, which enabled comprehensive scientometric visualization. Based on the research data collected, the following results were evaluated: Number of publications per year on Aflatoxin M_1_, countries’ scientific production, affiliation with aflatoxin research, most influential journals, and top globally cited papers from 2015 through to February 2025, in addition to a keyword co-occurrence map generated using VOSviewer. This bibliometric analysis has several inherent limitations. First, only articles published in the English language were included, which may have led to the exclusion of relevant studies published in other languages and introduced potential regional or linguistic bias. Second, the analysis relied on a single bibliographic database (Scopus), which, despite its broad coverage, may not comprehensively capture all research outputs related to AFM_1_. As a result, some relevant publications indexed in other databases may have been omitted.

### 2.2. Bibliography Analysis

To gain a comprehensive understanding of the scientific data collected on aflatoxin M_1_ in milk and dairy products, a bibliographic analysis is performed on different aspects, such as publication trends, research productivity, and the evolution of AFM_1_ research in the last decade.

**Figure 1:**
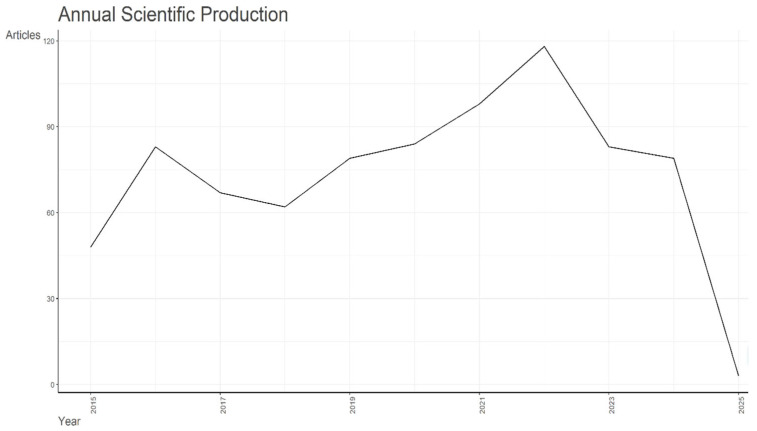
Number of publications per year on AFM_1_ research (2015–February 2025).

Figure 1 shows the scientific production related to aflatoxin in dairy products from 2015 to February 2025. The results reveal that the number of annual publications had increased gradually from 2018 to 2022, indicating a steady growth in research related to aflatoxin in dairy products. In addition, the number of publications increased steadily since 2018, leading to the largest number of publications in 2022, with 118 documents published. However, this apparent rise should be interpreted cautiously, as variations in publication volume, especially during 2020–2022, may reflect delays associated with the COVID-19 period rather than a sustainable growth trend. However, a decrease in document publication was observed from 2022 to 2023, followed by a stabilization at 79 documents published in 2024. This decrease observed in 2023 and the stabilization in 2024 likely represent a normalization in publication rather than a true decline in research activity. In addition, since the dataset is exclusively derived from Scopus, the observed trends are indicative rather than comprehensive. Furthermore, counts for 2024 and early 2025 represent incomplete publication years, as articles published later in the year were not indexed at the time of data extraction.

Table 1 highlights the top 20 contributing countries in AFM_1_ dairy research from 2015 to February 2025. Iran is the leader (17%), followed by China (11%), Brazil (8.2%), the United States (6.7%), and Egypt (6.6%). Many of the publications in the field of aflatoxin in dairy products research were published in Iran [32,33,34]. Iran might have prioritized the research on aflatoxin since it affects public health. Many regions in Iran have warm and humid weather, which favors the proliferation of the *Aspergillus* species, leading to a higher risk of aflatoxin contamination of animal feed and eventually in dairy products [35]. In fact, aflatoxin can cause many health problems, including liver cancer. Iran has made a lot of effort to address these concerns, which explains the number of publications. Outbreaks of acute aflatoxicosis may explain their alertness to this issue [36,37]. Iran’s contribution to the publication shows a national research effort and collaboration with an international organization focusing on public health and agricultural safety. On the other hand, China ranks as the second most prolific contributor, given its large agricultural sector and extensive maize production, which increases vulnerability to aflatoxin contamination entering the food chain and affecting milk and dairy products. The high number of publications can be explained by the rise in liver cancer, especially hepatocellular carcinoma linked to aflatoxin contamination, which made China intensify its research in this field [38,39]. The Efforts of Chinese authorities, in particular the National Natural Science Foundation of China (NSFC), may have led to an increase in funding for mitigation strategies for aflatoxin contamination in food products [40]. Brazil also represents a major contributor, which is consistent with its role as a global agricultural powerhouse. Aflatoxin contamination is an issue in Brazil, mainly in maize and peanuts. While maize constitutes a major component of animal feed, peanut by-products, such as peanut meal, are used as protein ingredients in animal feed in certain regions. Contamination of these feedstuffs with AFB_1_ is of particular concern due to its metabolic conversion to AFM_1_ in lactating animals and its excretion into milk. Since Brazil is a major exporter of agricultural products, there should be an increased interest in research to mitigate the aflatoxin contamination [41]. The United States is well-known for its interest in food safety research and regulations, mainly due to institutions such as the Food and Drug Administration (FDA) and the United States Department of Agriculture (USDA). Since it is one of the largest corn producers globally, the United States faces the challenge of AFB_1_ contamination in maize, which is a major component of animal feed and the primary source of aflatoxin M_1_ carry-over into the milk [42]. While aflatoxin outbreaks are less prevalent than in developing countries, the economic importance of corn has driven continuous work on detection techniques and consistent monitoring. This explains the steady contribution of the United States to research in the AFM_1_ field. On the other hand, Egypt also demonstrates a research contribution, indicating an increased awareness to address aflatoxin contamination. Given its reliance on agriculture and its warm climate, aflatoxin research may be an essential area for the country’s agricultural scientists. In fact, research assessments have shown many contaminations among dairy samples [43,44], which can explain its role in the research.

In addition to the geographic distribution of publications presented in Table 1, the analysis of institutional affiliations further clarifies the landscape of AFM_1_ research. Interestingly, the countries with the highest number of publications (Iran, Brazil, Pakistan, and China) also show the leading institutions contributing to this field. Table 2 presents the top 10 institutions based on the number of articles published between 2015 and February 2025. The analysis of institutional affiliations reveals the University of Sao Paulo as the most active institution in publishing documents on AFM_1_ research (8.9%), followed by Islamic Azad University (8.6%), Bahauddin Zakariya University (8.4%), and the Institute of Animal Science (6.8%), reflecting the strong research engagement of these countries, Brazil, Iran, Pakistan, and China, respectively, in securing public health. Many of these institutions are specialized in agriculture, medical medicine, and veterinary fields, showing the interdisciplinary nature of aflatoxin research.

The top 10 journals that have published on aflatoxin in dairy products are listed in Table 3. The data shows that *Food Control* is the most active journal, publishing the highest number of articles (76, 9.4%), followed by *Toxins* (55, 6.8%) and *Food Chemistry* (30, 3.7%). The top 10 journals with the greatest contribution to aflatoxin in dairy research account for 32.43% of all publications included in this study, highlighting their significant role in disseminating research on this topic. Several of these journals also demonstrate strong scientific influence, as reflected by their 2024 Impact Factors and CiteScores. *Food Chemistry* holds the highest impact factor (IF) (9.8) and CiteScore (18.3), confirming its status as a leading high-impact journal in food science. Similarly, *Food Control* (IF 6.3, CiteScore 14.1) and the *Journal of Dairy Science* (IF 4.4, CiteScore 7.8) show high citation performance.

The analysis of the 10 most-cited publications from 2015 to August 2025 revealed that journal activity does not always correlate with scientific impact (Table 4). 

Environmental Research and Public Health, which is not listed among the most active journals, published the most highly cited article: Alshannaq et al.’s [45] “Occurrence, Toxicity, and Analysis of Major Mycotoxins in Food”, with 919 citations. Likewise, Marchese et al. [46] and the European Food Safety Authority (EFSA) Panel on Contaminants in the Food Chain [47], who published in Toxins and the EFSA Journal, respectively, have high citations of 392 and 325 citations, emphasizing their influence in risk assessment and toxicological studies. Other highly cited articles, such as Flores-Flores et al. [50] and Iqbal et al. [51], focused on AFM_1_ in dairy, aligning with this study. Citation patterns reflect the complexity of research influence beyond journal activity. Even though the top listed journals, *Food Control* and *Toxins*, published the highest number of articles, the most influential research appears among various journals. This shows that citation count can be influenced by other factors, such as global research priorities, methodology, and study relevance, instead of journal frequency alone. Notably, open-access journals often achieve higher citation rates due to their broader visibility and unrestricted accessibility. In addition, the highest-rated articles are review articles, suggesting a high demand for a comprehensive overview of mycotoxin research. Reviews attract citations due to the greater coverage of different areas within the topic. Although these results cannot determine the quality or impact of a study, the citation count can serve as an indicator of a publication’s relevance. The top articles provide researchers with a solid foundation to identify knowledge gaps, current trends, and future research directions in AFM_1_, especially in detection methods, mitigation strategies, and regulations for aflatoxins in dairy.

Numerous authors, including Corassin, Oliveira, Zhang, Ismail, and Wang, emerged as significant contributors in AFM_1_ research. Some of them also feature in highly cited publications, indicating a correlation between research productivity and impact. Many of their works appear in high-impact journals such as Food Control, Toxins, and the Journal of Dairy Science, highlighting their ongoing effort in advancing the aflatoxin research field [53,54].

**Figure 2:**
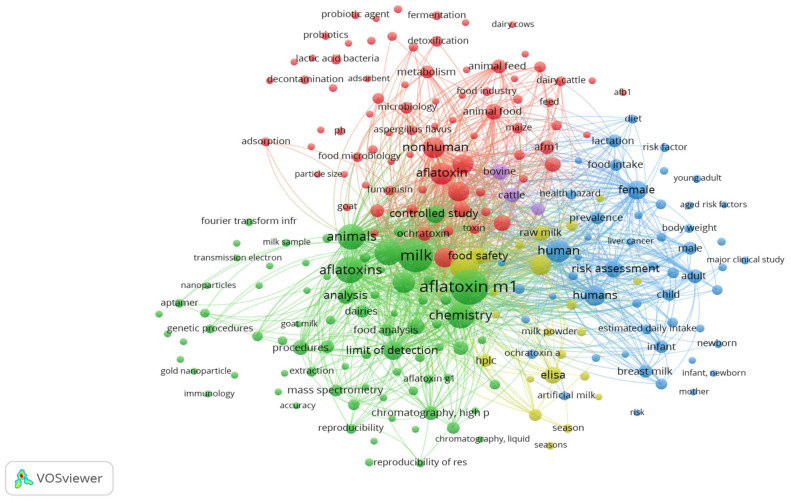
Keyword co-occurrence map of aflatoxin research in milk and dairy products generated using VOSviewer (version 1.6.20). Node size indicates the frequency of keyword occurrence, while colors represent clusters of related research topics.

A co-occurrence network of keywords was generated using VOSviewer to visualize the thematic structure of aflatoxin research in milk and dairy products (Figure 2). The analysis identified five major clusters, each representing a fundamental area within the literature:

1. The purple cluster with keywords such as bovine and cattle focuses on the carry-over of AFB_1_ from contaminated feed into milk in dairy livestock. This area of study is principal for understanding the biosynthesis of AFM_1_, which occurs in the liver of lactating animals after the ingestion of contaminated feed and is excreted into the milk. The finding shows the important role of the ruminants in the aflatoxin transmission chain;
2. The blue cluster highlights human health and risk assessment, including terms like risk factor, infant, female, liver cancer, breast milk, and estimated daily intake. This cluster focuses on AFM_1_ exposure, especially in vulnerable populations (infants, newborns, and breast milk), and its potential carcinogenic impact (health hazard, liver cancer);
3. The yellow cluster relates to prevalence studies, food safety monitoring, and exposure assessments with keywords such as food safety, season, Enzyme-Linked Immunosorbent Assay (ELISA), and milk powder. These terms indicate the application of analytical methodologies in prevalence studies, seasonal monitoring, and regulatory surveillance to ensure public health protection.
4. The green cluster centers around analytical methodologies in detecting AFM_1_ in dairy products, with terms such as chromatography, High-Performance Liquid Chromatography (HPLC), mass spectrometry, limit of detection, analysis, extraction, and nanoparticles.
5. The red cluster focuses on mitigation strategies targeting aflatoxin contamination in the pre-harvest and feed to food contamination, with terms like lactic acid bacteria, probiotics, fermentation, physicochemical methods, adsorbent, detoxification, and decontamination. These keywords indicate a strong research focus on mitigation, especially environmentally friendly methods, and food-grade interventions to bind, degrade, or eliminate aflatoxins either in animal feed or during dairy product processing. The presence of other terms such as animal feed, goat, and dairy cattle indicates, as well, the focus on limiting AFB_1_ exposure at the farm level to reduce AFM_1_ excretion into milk.

Finally, the central keywords, like aflatoxin M1, milk, and aflatoxins, appear as large nodes, indicating their high frequency and centrality within the field. All these keyword networks reaffirm the complex and interdisciplinary nature of AFM_1_ research and help guide more future studies related to mitigation, exposure assessment, and food safety. Guided by these bibliometric patterns and keyword clusters, the next section synthesizes evidence on AFM_1_ biosynthesis and toxicity in both animals and humans, and regulation, prevalence, and mitigation in dairy products.

## 3. Aflatoxin M1 Biosynthesis and Toxicological Impact on Animals

The presence of aflatoxins in milk and dairy products results from the contamination of animal feed, which serves as the primary source of aflatoxin exposure for animals [55]. The biosynthesis and survival of aflatoxin are influenced by many environmental and agricultural factors, such as temperature, relative humidity, pH value, substrate availability, and pre- and post-harvest handling [56]. Their stability and resistance to many processing methods pose a serious health threat throughout the food chain.

Under favorable conditions, such as warm temperatures ideally between 24 °C and 35 °C, relative humidity levels exceeding 50–60% [57,58], adequate oxygen availability, and high moisture content in grain (>13%), aflatoxin is produced and can contaminate crops, maize, grains, tree nuts, wheat, and other crops [59,60,61]. Relative humidity (RH) is a key constraint for fungal growth and aflatoxin production; however, reported RH thresholds differ across studies and commodities. Importantly, water activity (a_w_) represents the primary determinant of fungal growth and aflatoxin biosynthesis in foods and feed matrices, as it directly reflects the availability of free water within the commodity. While RH can be used as an indirect indicator of moisture conditions, its effect on aflatoxin production is mediated through its influence on the equilibrium water activity of the stored product [62]. Muga et al. [58] reported that storage at 60% relative humidity (RH), regardless of temperature, resulted in very low aflatoxin levels (<5 µg/kg), demonstrating the importance of controlled humidity to suppress fungal growth and toxin production. Moreover, guidelines published by the Food and Agriculture Organization (FAO) indicate that *A. flavus*, one of the fungi responsible for aflatoxin production, cannot survive at relative humidity below 70% and a temperature below 10 °C [63]. Aflatoxins can enter the feed chain at any stage from pre-harvest to post-harvest, with factors such as mechanical damage (grain breakage), improper drying post-harvest, environmental stressors (such as drought and excessive rain), insect infestation, competition with other microorganisms, application of pesticides and fungicides, and poor storage conditions (high humidity, high temperature, and inadequate ventilation) are critical in determining the extent of toxin accumulation [64,65,66]. The relative contribution of these factors varies substantially across agro-ecological contexts, explaining why aflatoxin prevalence remains heterogeneous, even within regulated systems. With the increase in global temperature and progression of climate change, regions that previously had minimal risk are now more vulnerable to *Aspergillus* growth and aflatoxin contamination [67].

Enzyme abbreviations: CYP450s, cytochrome P450 enzymes (mainly CYP1A2 and CYP3A4); GSTs, glutathione S-transferases; mEH/EPHX1, microsomal epoxide hydrolase; AFAR, aflatoxin aldehyde reductase; NADPH reductases, cytosolic oxidoreductases involved in aflatoxicol formation.

**Figure 3:**
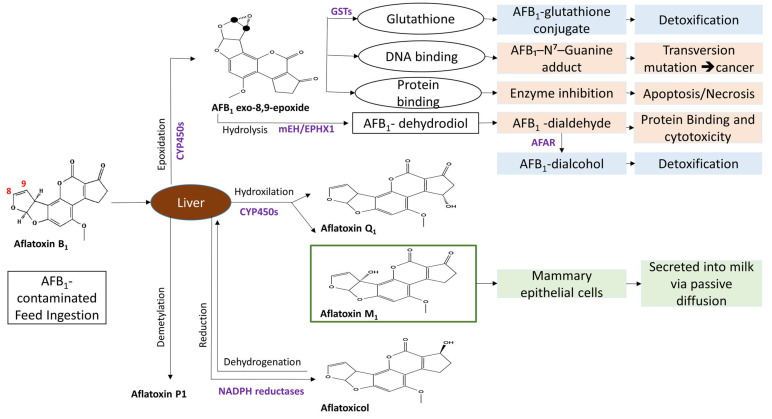
Metabolism of aflatoxin B_1_ and formation of aflatoxin M_1_ in lactating animals. In the liver, AFB_1_ is converted by Cytochrome P450 (CYP1A2, CYP3A4) to the reactive AFB_1_-8,9-exo-epoxide (AFBO), which can bind DNA or proteins (toxic pathways), undergo glutathione conjugation via glutathione S-transferases GSTs (detoxification), or be hydrolyzed by microsomal epoxide hydrolase mEH/EPHX1 to AFB_1_-dihydrodiol. The latter can form a toxic dialdehyde or be reduced to a detoxified dialcohol by aflatoxin aldehyde reductase (AFAR). Additional Phase I reactions yield AFM_1_, AFQ_1_, AFP_1_, and AFL (via NADPH reductases). In cows, AFM_1_ crosses mammary epithelial cells and is secreted into milk. Color legend: orange: toxic activation pathways (DNA binding, protein binding, and dihydrodiol/dialdehyde formation); blue: detoxification or non-epoxide metabolic pathways (glutathione conjugation, dialcohol formation, hydroxylation, and reduction); and light green indicates transport and secretion into milk.

When animals consume AFB_1_-contaminated animal feed, part of the toxin undergoes reversible reduction by the gut microbiome into aflatoxicol (AFL). The remaining AFB_1_ is absorbed in the small intestine by passive diffusion into the bloodstream and transported to the liver [68]. In the liver, members of the cytochrome P450 family (mainly CYP1A2 and CYP3A4) complete two main transformations: Hydroxylation at the 4-position converts it into the hydroxylated metabolite AFM_1_, a water-soluble molecule due to the hydroxyl group, which is excreted into the milk of lactating animals. Second, epoxidation at the 8,9-position produces AFB1-8,9-epoxide, the major carcinogenic metabolite of aflatoxin (Figure 3). The epoxide is very unstable and reacts with liver protein, DNA, and RNA, leading to tissue injury and liver toxicity. It forms DNA adducts at codon 249 of the p53 tumor suppressor gene, contributing to hepatocellular carcinoma development [46]. AFB_1_ exposure in animals is associated with liver damage, reduced animal feed intake, and reduced body weight, and can even lead to the death of the animal. AFM_1_ is eventually excreted in the milk of lactating animals, ultimately contaminating dairy products and posing a serious food safety concern [69]. Studies estimate that 0.3–6.2% of ingested AFB_1_ is converted to AFM_1_ [19,70,71]. In fact, just 6 h after animals are fed a diet with AFB1, AFM_1_ appears in milk [72]. The level of carry-over is determined by many factors, such as the animal species, the type of feed, the animal metabolism, animal breed, lactation period, detoxification ability in the animal liver, and milk production yield [19,71,73]. Seasonal variation that affects AFM_1_ levels in milk is attributed to changes in feeding practices. Higher concentrations of AFM_1_ are often seen in winter due to the reliance on stored feed, whereas lower levels are linked with grazing animals consuming fresh pasture [74,75]. In addition, the increase in rainfall and humidity during the winter season promotes fungal growth, further elevating the risk of animal feed contamination and eventually its presence in dairy products [76]. These observations underscore the importance of seasonal risk-based feed-monitoring strategies rather than uniform year-round surveillance.

Despite being present in trace amounts, aflatoxin poses a significant health concern due to its toxicity [72]. Since it is a small molecule, it is quickly absorbed in the digestive system. It targets the liver, leading to liver cancer [77]. Exposure to aflatoxin in dairy cattle affects animal reproduction, health, and productivity. Prolonged ingestion of contaminated animal feed can lead to reduced milk yield and low fat content [25], metabolic perturbation, and impaired liver, kidney, and lung function, [78]. A recent outbreak report from Pakistan showed that ingestion of concentrate feed contaminated with extremely high levels of AFB_1_ (165 μg/kg dry matter) resulted in a 75% abortion rate and an 18.8% reduction in milk yield in a dairy herd [22]. Immunity suppression increases the vulnerability to diseases [79] and impairs nutrient absorption, resulting in decreased growth rate [59]. Additional indicators include weight loss, diarrhea, hepatotoxicity, and nephrotoxicity [66]. Furthermore, the toxicity is increased by a combination with other mycotoxins that can be present in the environment, such as citrinin, as reported by Penagos-Tabares et al. [22], reinforcing the need for feed safety controls. Flores-Flores et al. [50] reported that milk from animals fed on grazing contains lower AFM_1_ content compared to those consuming stored or compound feed, supporting pasture-based feeding as a protective factor where climatic and management conditions allow.

## 4. Aflatoxin M1 Biosynthesis and Toxicological Impact on Humans

Human exposure to aflatoxins occurs directly through the consumption of contaminated agricultural products such as maize, wheat, and nuts, where AFB_1_ is the most prevalent toxin [51,80]. Inhalation of dust or fungal spores containing mycotoxin is another exposure route, though less common [81]. AFB_1_, the precursor of AFM_1_, is highly toxic and carcinogenic. In animals, ingested AFB_1_ is metabolized in the liver to AFM_1_, which is subsequently excreted into milk and dairy products. Humans are, therefore, indirectly exposed to AFM_1_ through the consumption of these contaminated animal products [51,80]. Exposure to aflatoxins may result in aflatoxicosis, with effects ranging from acute gastroenteritis to chronic diseases, depending on the duration and level of exposure. Acute aflatoxicosis results from ingesting highly contaminated food and causes gastroenteritis, acute hepatic necrosis, and death in severe cases. An acute aflatoxicosis outbreak occurred in humans in Kenya in 2004 due to consumption of maize highly contaminated with AFB_1_, resulting in 317 cases and 125 deaths [82]. In contrast, chronic aflatoxicosis occurs through long-term exposure to lower levels of aflatoxin, resulting in hepatocellular carcinoma, reproductive toxicity, immunosuppression, genotoxicity, nephrotoxicity, and impaired growth [83,84].

Despite having a carcinogenic potency at 2–10% of AFB_1,_ AFM_1_ is classified as a Group 1 human carcinogen by IARC due to sufficient evidence of carcinogenicity. AFM_1_ retains cytotoxic, mutagenic, and genotoxic characteristics [46,47]. In vitro, AFM_1_ exposure has been demonstrated to increase the generation of reactive oxygen species (ROS), a principal contributor to oxidative stress. This can lead to cellular damage and inflammatory responses [85]. In addition to oxidative stress, in vivo exposure to AFM_1_ in mice led to liver and kidney injury, reduced cell proliferation, and genotoxicity. Mechanistically, molecular docking analyses indicated that AFM_1_ can bind to DNA, histones, and tubulin, contributing to its genotoxic and cytotoxic effects [86].

AFM_1_ can also induce DNA damage and epigenetic alterations, including changes in methylation patterns. The coexistence of AFM_1_ with other mycotoxins, such as fumonisin (FUM), ochratoxin A (OTA), or Zearalenone (ZEN), may enhance the overall toxicity result of an exerted synergistic effect [87]. Abdallah et al. [6] reported the synergistic effect of AFM_1_ and microcystin-LR (MC-LR), a hepatotoxin produced by certain cyanobacteria, both carcinogenic hepatotoxicants. Their mixture led to a significant mitochondrial dysfunction, principally among the vulnerable population.

Due to their high milk consumption, children are especially vulnerable to AFM_1_ exposure. A risk assessment study in Ireland showed that children aged 8 to 12 years often exceed safety thresholds based on margin of exposure (MOE) values [25]. Moreover, a study in Malawi detected AFM_1_ in 98% of all milk samples, exceeding EU limits. While the estimated risk of hepatocellular carcinoma (HCC) was low (0.038–0.023 cases per 100,000 people per year), the study stressed the impact of AFM_1_ in hepatitis B virus (HBV) on endemic populations and vulnerable age groups. Aflatoxin and HBV infection act synergistically, increasing the risk of liver cancer, even at low doses of AFM_1_ [88].

Early-life exposure to AFM_1_ can occur through both breast milk and infant formula. Surveys of commercial infant formulae and breast-milk samples conducted in different regions consistently report detectable AFM_1_ levels in a high percentage of samples, even when concentrations remain below international safety limits [23,89,90,91]. Breastfed infants are, therefore, also at risk due to AFM_1_ carry-over into human milk. Exposure to contaminated breast milk has been associated with growth impairments and neurodevelopmental effects, in addition to a possible association between AFM_1_ exposure and autism spectrum disorders [92]. Hsu et al. [93] conducted a review of epidemiological studies and identified different results regarding AFM_1_ association with stunting. In addition, AFM_1_ has been linked to intestinal barrier dysfunction and disruption in insulin-like growth factor (IGF) signaling. These factors inhibit cell growth, division, and physical development, especially in children.

Multiple studies have documented AFM_1_ contamination exceeding regulatory limits worldwide: In southern Ghana, more than 55% of milk samples were contaminated with AFM_1_ exceeding regulatory limits. Although cancer risk was low (less than 0.07 cases of cancer per 100,000 people per year), the low margin of exposure values indicates chronic risk, especially among the vulnerable population [94]. Similar findings were published in Serbia, where domestic cheese products showed higher AFM_1_ levels, with margin of exposure values for preschool children indicating potential concern [95]. Another study in Kenya estimated hepatocellular carcinoma incidence due to AFM_1_ exposure in milk at 1.4 × 10^−3^ to 3.5 × 10^−3^ per 100,000 people/year, with the highest burden in children under five [96]. On top of that, a meta-analysis conducted in Iran assessed AFM_1_ concentration in various milk samples and found a consistent violation of regulatory limits. The carcinogenic risk was the highest for individuals under 20, mostly influenced by AFM_1_ concentration, indicating milk as the major route of exposure [97].

## 5. International Regulations of Aflatoxin M_1_

Aflatoxin cannot be completely removed from animal feed or the human diet, but its level can be reduced through the implementation of regulations and monitoring plans that set permissible limits and ensure compliance. Various countries and international bodies have established regulatory standards to control AFM_1_ levels in milk and dairy products. The maximum permissible limit for AFM_1_ varies across countries depending on several factors, such as economic development, political priorities, toxin data available, and analytical capabilities. Developed countries have established maximum permissible limits for AFM_1_, while most developing countries rely on standards set by international agencies [98]. In the United States, the Food and Drug Administration (FDA) sets a maximum limit of 0.5 ppb (0.5 µg/kg or 500 ng/kg) for AFM_1_ in milk, as indicated in Compliance Policy Guide Sec. 527.400 [99]. The limit for feed intended for dairy animals is 20 μg/kg of AFB_1_ [100]. Similarly, the Codex Alimentarius Commission, established by the Food and Agriculture Organization (FAO) and the World Health Organization (WHO), has also set a maximum limit of 0.5 μg/kg for AFM_1_ in milk [101]. This standard aims to protect public health and facilitate international trade. Many countries, especially those aligned with Codex Alimentarius recommendations, have adopted this limit, such as Brazil [102], China [103], India [104], and Ghana [105], along with other Asian nations.

However, the European Union has published stricter regulation limits. According to Commission Regulation (EU) 2023/915, the maximum levels for AFM_1_ are set at 0.05 μg/kg (50 ng/kg) for raw milk, heat-treated milk, and milk used for the manufacture of milk-based products [106]. While cheese does not have a specific AFM_1_ limit, its safety is indirectly regulated through the limits applied to milk, its primary raw material. For infants and young children, for formulae and milk-based products, the limit is even lower, at 0.025 μg/kg [106]. This low limit indicates a preventive strategy for food safety for the susceptible population. Many countries, such as Iran [32] and Turkey [107], have adopted limits aligned with the European Union’s maximum level for AFM_1_ in milk.

These differences between the regulations are due to the diverse risk assessment methodology used, which takes into account factors like public health impact, economic implications, and the socio-economic situations. The Joint Committee between FAO and WHO Expert on Food Additives (JECFA) conducted scientific evaluations in 2001, determining that there is no significant difference in public health outcomes between the 0.05 and 0.5 µg/kg levels. Furthermore, studies show that excessive regulations of AFM_1_ in milk may have unexpected consequences in food-insecure areas, where strict enforcement could limit the availability of milk and divert the focus from more critical challenges, such as aflatoxin B_1_ exposure [108]. In developing countries, enforcement remains weak and compliance is irregular. Strict legislations are frequently applied solely to export commodities, while local markets lack monitoring infrastructure [109]. These inconsistencies are caused by many factors, including poor feed storage practices, lack of monitoring, low awareness among farmers, inadequate regulatory enforcement, and climatic conditions that favor fungal growth [109].

Even with very strict regulations, the complete elimination of AFM_1_ in milk is not achievable due to many factors, such as environmental contamination, limitations of detection methods, and mitigation technologies. The strict regulation reduces, but does not eliminate, the exposure to mycotoxins or the synergistic effects with other mycotoxins. Therefore, a combination of different methods, whether educational, regulatory, or agricultural, remains important for effective aflatoxin control in the dairy sector.

## 6. Global Prevalence of Aflatoxin M_1_

The presence of AFM_1_ in milk and milk products is frequently reported throughout the world. The prevalence of AFM_1_ in milk is partially attributed to the lack of knowledge among farmers and contaminated feed, in addition to unsafe storage conditions [110]. Market prevalence data gathered from different countries highlight a vast variation of AFM_1_ concentration in different dairy products. Table 5 shows that countries with established monitoring systems, such as Italy, Spain, Ireland, and France, generally report low-to-non-detectable levels of AFM_1_. For example, as seen in Table 5, a study in Ireland showed that AFM_1_ concentration in milk ranges between 0.00087 and 5.72 ng/L, which is below the European Union’s permissible limit of 50 ng/L [25]. Similarly, research in Spain found no samples (full-cream or raw milk) above the detection limit [111]. These results show the importance of constant monitoring, good agricultural practices, and enforced regulations in European countries. In contrast, some African countries report alarming levels of AFM_1_ in milk and dairy products: in Nigeria, AFM_1_ concentrations reached 3108 ng/L in goat milk, with 55% of samples exceeding the regulatory limit, while cow milk reached up to 81 ng/L [112]. Moreover, in Ghana, 94.6% of cow milk samples were contaminated with AFM_1_, with concentrations ranging from 61.8 to 1606.8 ng/L, exceeding the EU limit in all tested samples [105]. Additionally, 82.4% of cow milk samples were contaminated with AFM_1_ in Sudan, with 47.1% of the samples ranging between 100 and 150 ng/kg [113]. These numbers show an alarming risk to public health, especially in rural communities where raw milk can be consumed without adequate safety controls. Other countries, such as Bangladesh, Egypt, and Iran, showed high AFM_1_ contamination across different dairy products. For example, Ewida et al. [114] reported a very high level in a traditional Egyptian fermented cheese, Mish cheese, reaching 40,500 ng/kg, a level exceeding EU and US standards. Egypt reports some of the highest contamination levels in various dairy products [43,44,114]. Another study in Bangladesh reported contaminated raw milk with AFM_1_ reaching 1489.28 ng/kg [115]. These results can be linked to different factors, such as animal feed storage conditions at the farm, a lack in enforcing regulations, climatic conditions favoring the growth of the fungus, and economical constraint. In Lebanon, AFM_1_ contamination has also been documented. A recent study reported AFM_1_ levels ranging between 10.7 and 440 ng/L in cow milk, with approximately 36% of samples exceeding the EU limit [116]. Earlier baseline monitoring had also shown 21% of dairy products above the EU maximum level and higher concentrations in soft cheeses compared to milk [117]. Therefore, it is important to have strict regulations and control measures, including routine animal feed testing, cold chain infrastructure, and a farmer training schedule, given the chronic exposure effect of aflatoxins [118]. Surprisingly, some countries, despite having regulated limitations, continue to report high contamination levels in dairy products. For example, in Italy, Roila et al. [119] found AFM_1_ in 2244 out of 3151 of cow milk samples, with concentrations reaching 146 ng/kg. This shows that even if regulations are present, enforcement remains challenging. On top of that, the impact of climate change in different regions also plays a role in the occurrence of AFM_1_. Areas with hot and humid climates, such as Egypt, Bangladesh, and Iran, have favorable conditions for *Aspergillus* species growth, thus increasing aflatoxin contamination levels in animal feed and, eventually, in milk. In addition, AFM_1_ concentration can be influenced by different processing methods used in dairy products across different countries. AFM_1_ binds casein and therefore partitions into curd at coagulation, so cheeses, especially rennet-set, tend to show higher concentration factors than the source milk. During brining or ripening, a fraction may diffuse to whey or brine, while fermentation can yield modest reductions through microbial activity [120,121]. Furthermore, the difference in analytical methods used, ELISA, HPLC, or Liquid Chromatography–Tandem Mass Spectrometry LC-MS/MS, can lead to inconsistency in detection sensitivity and quantification accuracy.

## 7. Mitigation Strategies

The presence of AFM_1_ in milk is a serious food safety threat. Knowing the right and effective method to reduce it will decrease and prevent economic losses, preserve milk safety, and protect public health. Several mitigation strategies to reduce AFM_1_ are available, whether directly targeting the milk or indirectly targeting the animal feed or the animal itself. These methods can be classified into biological, chemical, and physical techniques (Table 6). Table 6 provides a comprehensive overview of the main mitigation strategies investigated for reducing aflatoxin B_1_ (AFB1) in feed and aflatoxin M_1_ (AFM_1_) in milk and dairy products, summarizing their reduction efficiency, principal advantages, and key limitations, and highlighting the trade-offs between effectiveness, safety, regulatory acceptance, and industrial applicability across chemical, biological, physical, and integrated approaches.

### 7.1. Chemical Mitigation Techniques

Several chemicals have been studied for their ability to detoxify aflatoxins. These include alkaline agents, oxidizing agents, acidic agents, and inorganic adsorbents, among others (Supplementary Table S1). Their mechanisms of action vary from degrading, transforming, or binding the toxin to reduce its availability and toxicity [142].

#### 7.1.1. Alkaline Agents

**Figure 4:**
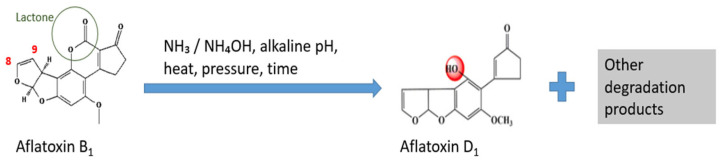
Ammoniation of aflatoxin B_1_ (AFB_1_) and formation of aflatoxin D_1_ (AFD_1_).

Ammonization of feed is a technique using an alkaline agent to detoxify AFB_1_ contamination in animal feed. Under alkaline conditions, ammonia induces nucleophilic attack on the lactone carbonyl and the difuran double bond of AFB1, triggering alkaline hydrolysis. This reaction opens the lactone ring and disrupts the 8,9-double bond, preventing the formation of the toxic AFB1-8,9-epoxide. The toxin is converted into less harmful products, such as aflatoxin D1 (AFD1), aflatoxin D2 (AFD2), and related derivatives that show reduced mutagenicity because they can no longer form DNA adducts (Figure 4) [143,144,145]. Reversion to AFB1 can occur under acidic conditions, when treatment is insufficient [143]. Ammonia gas or ammonium hydroxide is applied to contaminated animal feed under controlled environmental conditions, including pressure, temperature, and duration [145]. Studies have shown that ammonization can reduce AFB_1_ levels by 70 to 90% due to the instability of AFB_1_ under alkaline conditions [143,146]. This technique has been successfully applied to many feedstuffs, such as cottonseed meal, peanut meal, maize, and others [146,147]. Furthermore, optimized ammonia treatment protocols, such as 4% NH_4_OH, 2 bar pressure, and 100 °C for 60 min, can reduce AFB_1_ levels by up to 96.3% in groundnut press cake [147]. Therefore, the degradation is shown to be effective under certain intrinsic or extrinsic conditions. As AFB1 loses its structural features required for hepatic hydroxylation to AFM1, ammonization significantly decreases its carry-over into milk, with a stated decrease of 30-fold in dairy cows [148,149]. However, this method has certain setbacks and safety concerns, such as a possible reduction in feed nutritional value, high implementation costs, and challenges in maintaining optimal processing conditions. In addition, ammonia handling safety and residue monitoring are important for approval and industrial-scale use [79]. Despite its high efficacy, ammonia treatment is primarily limited to feed decontamination due to its unsuitability for direct application to dairy products. Another limitation is the reliance on strictly controlled processing conditions, which makes it more applicable to industrial-scale feed processing.

#### 7.1.2. Oxidizing Agents

##### Ozonation

**Figure 5:**
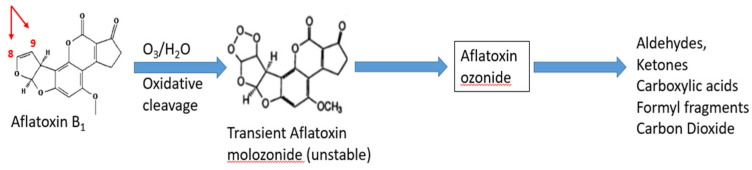
Ozonation mechanism on aflatoxins.

Ozonation is a non-thermal, residue-free chemical detoxification method, in which ozone (O_3_), a strong oxidizing agent, breaks down aflatoxins through electrophilic attack and oxidative reactions that cleave the C8 to C9 double bond in the furofuran ring [150]. This reaction disrupts the structure responsible for AFB1 and AFM1 toxicity, converting them into less harmful products, such as carboxylic acids, aldehydes, and smaller chain fragments (Figure 5) [151]. Because ozonation relies on chemical degradation, its efficiency depends on ozone concentration, exposure time, humidity, and matrix composition. In dairy matrices, ozone exposure can decrease AFM1 levels, although reductions remain moderate. Mohammadi et al. [152] reported a reduction of about 53% of AFM_1_ after 10 min of treatment. However, the treatment affected milk quality, such as altered color and texture, a decrease in β-carotene, and a lower microbial count. Sert and Mercan [153] demonstrated that exposure of milk and whey concentrates’ matrices to ozone for 60 min led to a decrease of 18.9% and 9.9% in AFM_1_ levels, respectively, confirming modest efficacy but improved microbiological quality without chemical residues. For feed detoxification, which indirectly lowers AFM_1_ in milk, higher efficiencies have been reported. Ozonation of corn grits (20–60 mg/L) with an exposure time of 480 min achieved a maximum AFB_1_ reduction of 57% [154]. In contrast, Rahmani et al. [155] optimized ozonation conditions for ground corn using 600 mg of ozone per kilogram of corn for 250 min, reducing AFB_1_ by up to 96 to 99%. However, the industrial application of this treatment may be limited by scalability, the need for high doses, and long exposure time to achieve the best reduction. Recent developments show the importance of hurdle-based approaches. Khoori et al. [156] studied the synergistic effect of different processes, such as the use of ozonation, ultraviolet radiation, and pulsed electric field techniques for reducing AFM_1_ in probiotic milk. The decrease in AFM_1_ reached 96.1% while maintaining a stable *Lactobacillus acidophilus* count of 10^6^ CFU/g. Optimal conditions for maximum AFM1 reduction included an ozone concentration of 9.99 mg/min, 13.15 microsecond pulse duration, and 4.99 J/cm^2^ UV intensity. Thus, indicating the importance of hurdle technology can compensate for the moderate efficacy of ozone alone. Overall, ozonation is considered environmentally friendly and residue-free. However, the moderate reduction in AFM_1_ and the potential sensory and nutritional alterations in milk limit its use [157]. Further research is needed to clarify this method’s stability and the toxicological safety of AFM1 ozonation by-products in complex dairy matrices before regulatory approval can be considered. Industrial feasibility remains constrained unless applied in combination with other physical processes within a hurdle-technology framework.

##### Hydrogen Peroxide

Hydrogen peroxide has also been studied as a method for mitigating AFM_1_ contamination in milk and animal feed. Its mechanism relies on the generation of reactive oxygen species (ROS), mainly hydroxyl and perhydroxyl radicals that cleave the C8–C9 double bond in the terminal furan ring of aflatoxins, leading to ring opening and conversion into smaller oxidized fragments with reduced toxicity. Applebaum and Marth [158] have shown that hydrogen peroxide in the presence of riboflavin and lactoperoxidase completely inactivates AFM_1_ (0.7–1.7 μg/L) in raw milk. Another study combined hydrogen peroxide (0.5 g/L) with UV irradiation for 20 min in milk, eliminating 89% of AFM_1_ compared to 61% reduction with UV alone [159]. More recently, Shen and Singh [160] applied a high concentration of hydrogen peroxide (30% w/w) at 50 °C to peanuts, achieving up to 90% reduction in AFB_1_, with residual peroxide effectively removed by drying. In this study, post-treatment aflatoxin levels met U.S. FDA feed guidance, but not those for human food. Despite its effectiveness, hydrogen peroxide-based oxidative treatments can oxidize milk components, such as ascorbic acid, and certain amino acids, potentially altering its sensory and nutritional quality [161,162]. Higher concentrations in food can be a risk to human health, causing serious gastrointestinal problems [163]. Although, hydrogen peroxide can be effective when used in combination with other techniques such as UV irradiation, its industrial feasibility in dairy remains limited due to safety and regulatory constraints [164].

#### 7.1.3. Adsorbents

##### Bentonite

Bentonite is an aluminosilicate clay mineral with a negatively charged layered structure and a high cation-exchange capacity [165]. Its mitigation mechanism is physical adsorption, thus binding aflatoxins and decreasing their bioavailability in dairy products and animal feed [166]. Aflatoxin molecules are immobilized on clay surfaces and in interlayer spaces through electrostatic attraction, hydrogen bonding, Van der Waals forces, and cation interactions [167,168].

Bentonite has reduced aflatoxin when incorporated into animal feed. Dietary supplementation with bentonite and montmorillonite reduced AFM_1_ levels in goat milk by approximately 64.5% and 82.2%, respectively [169]. Similarly, bentonite has been shown to adsorb between 90.0% and 95.3% of AFB_1_ at pH 5, indicating a solid binding capacity under acidic conditions, especially under ruminal and gastrointestinal conditions [165]. In field conditions, calcium montmorillonite-based toxin binders, such as NovaSil^®^, were introduced to animal feed, reducing the AFM_1_ contamination level in milk by about 90% and improving yield [170], while purified NovaSil Plus decreased AFM_1_ by 55–68% without altering milk composition or vitamin content [171].

The safety and efficacy of bentonite have been confirmed in dairy cows. Supplementation of Holstein cows with 2% montmorillonite-rich clay lowered AFM1 excretion from 27.81 to 16.51 μg/day and reduced transfer rate from 1.37% to 0.74%, although slight reductions in milk yield and changes in liver enzymes were reported, indicating the importance of dose optimization [78]. Additional trials reported reductions of 66.7% after 56-day supplementation, accompanied by improvements in serum protein, immunity, and milk production [172]. In vivo studies have shown bentonite’s efficacy in reducing aflatoxin. Kihal et al. [173] compared several binders, including hydrated sodium calcium aluminosilicate (HSCAS), bentonite, yeast cell wall (YCW), and multi-toxin binder mixes (MX), and demonstrated that bentonite showed the highest reduction in AFM_1_ in milk, with a mean reduction of 40% compared with other adsorbents.

Various optimized versions of bentonite have been developed to improve its effectiveness. G.Bind, an Iranian, locally processed bentonite, reduced AFM_1_ levels in milk and decreased the AFB_1_ transfer rate from 1.17% to 0.39% [174]. Likewise, size-fractioned bentonite particles (smaller than 5 µm) were shown to reduce the carry-over rate of 0.43%, which is comparable to commercial alternatives [175]. Recently, Ibrahimi Khoram Abadi and Heydari [176] found that magnetic bentonite nanocomposites (MBNC) exhibited the lowest AFM_1_ carry-over rate, along with a better milk yield and quality in Baluchi ewes, surpassing both natural and conventional modified bentonite.

Combining bentonite with microbial agents has increased AFM_1_ reduction. Bentonite-activated carbon mixture with non-viable *Bacillus coagulans* and *Lactobacillus paracasei* achieved complete AFM_1_ removal from milk spiked at 0.2 µg/L. The mixture remained stable through repeated washing cycles [177]. Other combinations with acid-killed or heat-killed lactic acid bacteria achieved a reduction of 97.6% [178]. Therefore, synergistic formulations can improve the mitigation results considerably compared to bentonite alone.

Although not yet approved for industrial milk processing, in vitro studies show high adsorption potential. Ca-bentonite removed up to 97.7% of AFM_1_ in artificially contaminated raw milk without nutritional losses [179]. Similarly, HAFR 3, a bentonite variant, removed 98.5% of AFM_1_ in milk within 12 h, with slight alteration to the milk nutritional values [166]. In another study, bentonite and date pit reduced AFM_1_ by up to 68% and 56%, respectively, among the adsorbents tested [180].

Given its wide use in animal feed, bentonite safety has been assessed. The European Food Safety Authority (EFSA) evaluated the safety of bentonite as a feed additive in 2011 and again in 2017. Findings concluded that bentonite is safe for all animal species when used at levels up to 0.5% in complete feed, and up to 2% (20 g/kg) for specific applications, with no adverse effects on consumers, animals, or the environment. However, due to its silica content, bentonite is considered a respiratory hazard for users handling it in its dry form. In the United States, calcium montmorillonite bentonite (marketed as NovaSil^®^) has been recognized by the Food and Drug Administration (FDA) as Generally Recognized as Safe (GRAS) for use in animal feed [165,181]. However, direct addition to milk is not authorized for food processing. Thus, bentonite is feasible at an industrial scale, but only as a feed additive, not as a milk decontaminant.

In comparison to commercially available binders, bentonite is regarded as a more cost-effective alternative and has demonstrated safety, particularly in relation to compliance with regulatory approval criteria. However, other commercial products with multi-component formulations offer a broader binding spectrum. For example, yeast cell wall-based Mycosorb^®^ reduced AFM_1_ secretion by 47% in buffaloes and improved milk composition [182]. Similarly, Mycofix^®^ (a blend of enzymes and adsorbents) reduced AFM_1_ levels by 22 µg/day and lowered the carry-over from 4.60% to 3.44% [183]. These findings accentuate the diversity of binding strategies beyond clay-based materials.

Bentonite and its derivatives are among the most researched binders for aflatoxin detoxification in animal feed. Its use has proven to be effective, safe, and accepted by regulatory bodies. However, results vary depending on bentonite particle size, formulation, and co-supplementation strategies.

##### Other Dietary Adsorbents

Other dietary adsorbents (yeast-based formulations, composite binders, natural aluminosilicates, and emerging engineered nanostructured materials) reduce AFM_1_ contamination in milk indirectly by adsorbing AFB1 in the gastrointestinal tract, limiting its absorption and biotransformation to AFM_1_. The effectiveness of these binders is affected by their composition, physicochemical characteristics, and levels of contamination.

Composite binders have demonstrated varying levels of effectiveness depending on the degree of aflatoxin exposure. Solis Mos^®^, a composite binder containing sodium montmorillonite, mannan oligosaccharides (MOS), yeast culture, and vitamin E, was found to be effective at moderate aflatoxin contamination levels [184]. In cows fed 20 µg/kg AFB_1_, the binder reduced AFM_1_ concentrations in milk by 16%, total excretion by 18.3%, and the feed-to-milk transfer rate by 17.9%. However, when the contamination level is high (40 µg/kg), no significant reduction was observed. This shows that certain binders might be ineffective when exposed to high levels of toxins, especially in areas where aflatoxin outbreaks occur frequently [184].

Natural aluminosilicate-based adsorbents, such as Clinoptilolite, also demonstrated efficacy. In their study across 15 commercial farms, Katsoulos et al. [185] reported that dietary supplementation of 200 g/day of Clinoptilolite with a smaller particle size (<0.15 mm) reduced AFM_1_ concentrations by 56.2%. These findings show the role of particle size optimization in enhancing adsorption, which is consistent with results from processed bentonite forms.

Another strategy is the use of blended mineral–organic formulations. Cha et al. [186] tested two dietary adsorbents: AD1, a combination of montmorillonite and diatomite (50:50), and AD2, a commercial product composed of montmorillonite, diatomite, yeast cell-wall extracts, and sodium alginate. Both were administered at 15 g/day to cows exposed to a moderate AFB_1_ level (8 µg/kg). Both products demonstrated AFM_1_ reduction in milk (from 93 ng/L to 46 ng/L and 51 ng/L, respectively) and decreased the carry-over rates (1.16% to 0.57% and 0.63%, respectively). The combination of mineral adsorbents with organic components, such as polysaccharides or cell-wall fragments, improved AFM_1_ building within the gastrointestinal tract.

Moreover, Costamagna et al. [187] evaluated Antitox CooPil^®^, which is composed of 60% zeolite and 40% cell wall, and demonstrated that supplementation reduced milk aflatoxin M_1_ concentrations from 0.016 µg/kg to 0.008 µg/kg and carry-over rates from 2.19% to 0.77%, even at low contamination levels. In parallel, advanced nanostructured materials have been developed to enhance adsorption efficiency. Moradian et al. [188] developed an engineered nanomaterial composed of mesoporous metformin–chitosan/silica-cobalt ferrite nanospheres (Mt-CS/CFS NSs), which demonstrated reduction in AFB_1_ by over 91% in milk under optimized conditions. Their mechanism involves multilayer adsorption, magnetic separation potential, and enhanced intermolecular interactions. These emerging materials are promising techniques for efficient and food-safe detoxification, especially in systems where conventional binders may be less effective.

Evidence from different studies showed that the efficacy of dietary adsorbents is dependent on formulation characteristics such as mineral type, pore structure, particle size, presence of organic additives, and the level and nature of aflatoxin exposure. The shift toward composite and precision-engineered materials suggests a direction toward more efficient binders. However, significant challenges remain regarding cost-efficiency, safety, and regulatory validation.

### 7.2. Biological Mitigation Techniques

Biological mitigation strategies are very promising approaches for reducing AFM_1_ contamination in dairy products because they offer specific, effective, cost-effective, and environmentally friendly techniques. These strategies often rely on enzymes, probiotics, and bioactive compounds that can either degrade, bind, or prevent the formation of the aflatoxin (Supplementary Table S2) [189].

#### 7.2.1. Microorganisms

##### Lactic Acid Bacteria

Lactic acid bacteria (LAB) are one of the most studied microorganisms for the detoxification of AFM_1_ in milk. Their detoxification activity is primarily attributed to non-covalent physical adsorption of AFM1 onto structural components of the bacterial cell wall, such as peptidoglycan, teichoic acids, surface proteins, and exopolysaccharides.

Various genera showed promising results in AFM_1_ binding in milk systems under different conditions, especially *Lactobacillus* [190]. *Lactobacillus* species can bind and reduce AFM_1_ levels in milk by a range of 20–100%, depending on strain, viability, temperature, exposure time, and matrix composition. For instance, *L. acidophilus* showed 71.46% reduction in AFM_1_ at 4 °C, and *L. casei* reached 64.31% at 37 °C, both inoculated in skim milk [191]. On the other hand, strains of *L. brevis* and *L. plantarum* isolated from traditional Iranian cheese reduced AFM_1_ by up to 50% over 72 h of refrigeration [192]. AFM_1_ detoxification does not require cell viability. In fact, heat-treated LAB cells are equally or even more efficient than viable ones: *Weissella confusa* H1 and *L. plantarum* S2 reduced AFM_1_ by 78% and 72%, respectively, in PBS buffer at 37 °C after 72 h, using both viable and heat-treated cells [193]. Muaz et al. [194] used acid-killed LAB in combination with a surfactant sorbitan monostearate, achieving 100% removal of AFM_1_ from skimmed milk spiked at 0.05 ng/mL.

Synergistic combination of strains can further enhance AFM_1_ removal. Yogurt with probiotics spiked with 20 ng/mL of AFB_1_ demonstrated binding efficiency of *L. acidophilus* PTCC 1643 (Persian Type Culture Collection) and *L. rhamnosus* PTCC 1637 between 64.56% and 96.58% [195]. LAB performance is strongly modulated by environmental conditions. High cell density, extended contact time, and active fermentation generally improve toxin removal, whereas binding may be reduced once fermentation is completed. *Streptococcus thermophilus* and *L. delbrueckii subsp. bulgaricus* showed high AFM_1_-binding capacity in milk samples (90–100%), but were ineffective in already fermented yogurt, since the bacteria are no longer active [196].

Beyond traditional experimental optimization, computational tools have been applied to optimize LAB performance in aflatoxin detoxification. Jafari et al. [197] used machine learning to model and predict optimal strain ratios, environmental conditions, and incubation times for AFM_1_ removal. The study demonstrated that *L. lactis* at 10^9^ CFU/mL reduced AFM_1_ in kashk by up to 70.91% after 5 days and by almost 72% after 30 days, with machine-learning predictions closely matching experimental observations. This demonstrates how computational tools can complement microbial detoxification techniques, opening new perspectives for precision in industrial dairy processing.

From an industrial perspective, LAB-based mitigation is advantageous because the microorganisms are already widely used in dairy fermentation, are safe (GRAS/QPS), and do not introduce chemical residues. However, several limitations must be acknowledged: adsorption is reversible, toxin desorption may occur under gastrointestinal or heat conditions, results from laboratory media may not translate to real dairy matrices, and variability among strains remains substantial [198,199,200]. Future research should select the most effective strain under realistic contamination levels, test its performance in industrial systems and in vivo, and develop effective synergistic probiotic formulations.

##### LAB and Yeast Synergies

Yeasts, such as *Saccharomyces cerevisiae* and *S. boulardii*, have shown promising results when it comes to AFM_1_ reduction, primarily through physical adsorption onto cell-wall components rich in β-glucans and mannoproteins [201,202]. *S. boulardii* (at 109 CFU/mL) alone showed a high AFM_1_ reduction of 96.88% in skimmed milk [191]. In Minas Frescal cheese, where the curd was originally spiked with 0.5 µg/kg with AFM_1_, *S. cerevisiae* alone achieved complete removal of AFM_1_ by day 20 of storage [200]. While yeasts alone show strong detoxification, synergies with LAB often accelerate the process. A combination of heat-killed LAB (*L. rhamnosus, L. lactis*) and yeast (*S. cerevisiae*) achieved the same results by day 10 of cheese maturation, twice as fast as yeast alone [203]. This reduction is mostly due to complementary adsorption sites provided by LAB peptidoglycans and exopolysaccharides. Recent evidence by Salem-Bekhit et al. [204] achieved even faster removal of AFM_1_ in milk by 24 h, using a combination of *L. rhamnosus* and *S. cerevisiae,* with an AFM_1_ reduction of 98.4%. Moreover, a probiotic combination of *L. rhamnosus, L. plantarum*, and *S. boulardii* also achieved 100% removal of AFM_1_ in skimmed milk spiked at 0.5 ng/mL, while *L. rhamnosus* alone achieved up to 91.82% reduction. These results highlight the complementary adsorption properties between LAB (peptidoglycan, teichoic acids, and EPS) and yeasts (β-glucans, mannoproteins), producing a broader range of binding sites and potentially more stable toxin immobilization. LAB–yeast combination is safe and used industrially. Future work should, therefore, assess adsorption stability during ripening and digestion, and evaluate the feasibility of standardized mixed-culture applications at an industrial scale [200].

##### Bifidobacteria and Cell-Wall Components

Bifidobacteria, commonly found in the gastrointestinal tract of humans and animals, are widely used as probiotics due to their health benefits. Their antimicrobial properties inspired their use in many dairy products, such as yogurt, cold desserts, buttermilk, and cheeses [205]. Research has explored their potential to mitigate AFM_1_ contamination through physical adsorption onto cell-wall polysaccharides, peptidoglycan, and other surface structures. Reported AFM_1_ reduction ranges from 20 to 96%, depending on strain, viability, dairy matrix, AFM_1_ concentration, and contact time [206]. For example, *Bifidobacterium* species, such as *B. bifidum*, achieved a reduction of 40.14% in cow milk and 42.9% in sheep milk [207]. While LAB and *Bifidobacterium* strains isolated from local dairy products demonstrate an antitoxin effect from binding AFB_1_ in contaminated PBS, many strains showed different AFB_1_ reduction rates, ranging from 12.1% after 6 h to 89.9% after 36 h of incubation at 37 °C, with longer contact times yielding greater toxin removal [208]. Importantly, cell viability is not essential; in several experiments, cell-wall fractions (postbiotics), including isolated peptidoglycan, outperformed live cells. Adácsi et al. [209] demonstrated that *Bifidobacterium animalis* BB12 and *L. lactis* R703 peptidoglycan isolates reduced AFM_1_ from skimmed milk by up to 42%. On the other hand, live cells achieved only up to a 21% reduction. These findings support the use of postbiotics in AFM_1_ detoxification, especially when viability is not desirable, such as with UHT products.

Despite their potential, Bifidobacteria-based detoxification is strain- and matrix-dependent. The stability of AFM1 binding during processing and digestion remains uncertain. Future work should evaluate standardized postbiotic formulations and validate their performance under realistic industrial conditions.

##### Synergistic Strategies

Synergistic approaches have been explored by combining probiotic strains with other agents, such as nanoparticles or surfactants, to enhance mycotoxin removal. Zamani et al. [210] used a newly isolated *Lactobacillus plantarum* strain from cheese combined with chitosan nanoparticles for AFB_1_ detoxification in vitro. Synergistic effects showed a reduction of 69%, suggesting its potential addition to animal feed as a toxin binder. For instance, yeast strains identified as *Saccharomyces genus* isolated from fermented products in combination with titanium dioxide nanoparticles showed a boosted reduction in AFB_1_ in the medium by 15 to 20% more than each treatment alone [211]. Further reduction in AFM_1_ was achieved when sorbitan monostearate was combined with acid-killed LAB, achieving a detoxification of 100% at lower toxin levels [194]. The latter study also found that removal of exopolysaccharides from cell surfaces further reduced binding efficiency, confirming the role of the cell wall in mycotoxin adsorption.

#### 7.2.2. Indirect Mitigation via Feed

Biological mitigation of AFB_1_ in animal feed is the most practical strategy for reducing AFM_1_ in milk. Unlike microbial adsorption in milk, these approaches rely primarily on enzymatic degradation of AFB_1_ within the feed, reducing the amount available for ruminal absorption and hepatic conversion to AFM_1_. *Bacillus subtilis* YGT1, isolated from yogurt, showed its ability to degrade 83.8% of aflatoxin B1 after 48 h of incubation, demonstrating its enzymatic ability to cleave the furan ring and convert AFB1 into less toxic metabolites [212]. Similarly, Guo et al. [213] evaluated the use of another strain of *Bacillus* isolated from fish gut, *Bacillus subtilis* ANSB060 biodegradation product (BDP). Findings showed that the addition of 0.2% BDP to an AFB_1_-contaminated feed reduced AFM_1_ concentrations in milk by 27% in Holstein cows and lowered the AFB_1_ to AFM_1_ transfer rate from 1.06% to 0.76%. This BDP did not affect feedstuff or milk production and composition. These strains are especially promising for on-farm applications, where dietary interventions are feasible. However, their efficiency depends on AFB_1_ contamination level, enzyme stability in feed matrices, and rumen conditions. Further research is needed to characterize degradation by-products, confirm their safety, and confirm efficacy across different feeding systems.

#### 7.2.3. Enzymes and Bioactive Compounds

The use of enzymes and bioactive compounds demonstrated promising results in AFM_1_ detoxification by breaking down the toxin’s chemical structure through oxidation or hydrolysis, by disrupting the C8=C9 double bond, or by modifying the furan ring, thereby reducing toxicity [2,214,215]. Some of these enzymes include superoxide dismutases (SOD_s_), peroxidases, lipases, and proteases, either in free form, immobilized, or expressed via recombinant technology. Enzymes such as protease and lipase produced by *Levilactobacillus brevis* and *L. plantarum* reduced AFM_1_ levels by up to 78.5% in contaminated yogurt over a period of five days of storage. However, these results are time-dependent and may be limited by the slow metabolic rate under refrigeration [214]. Recent advances in recombinant technology demonstrated higher efficiency of more than 60% AFM_1_ degradation using recombinant peroxidases (rPODs) in a model solution under specific conditions (pH of 9–10, temperature of 30 °C, and 60 mmol/L H_2_O_2_). However, when applied to milk, the degradation reached 25.6% due to the decrease in the enzyme activity under a more acidic medium [216]. Similarly, recombinant superoxide dismutase SODs removed more than 60% AFM_1_ in buffers but only 26.03% in the milk matrix limitations [2].

Immobilized enzymes have been applied for AFM_1_ detoxification in milk. Integrating enzymes with nanocomposite supports improved detoxification rates due to the enhanced enzyme stability. A nanocomposite composed of magnetic nanoparticles, chitosan, molybdenum disulfide, and laccase enzyme (Fe_3_O_4_/Cs/MoS_2_/Lac NPs) achieved 68.5% reduction in AFM_1_ after only 1 h of application to milk, demonstrating improved stability and reusability [217].

Physical activation methods can further increase enzymatic activity. Kerstner et al. [218] used peroxidases extracted from soybean meal and rice bran. In this study, ultrasound, microwave, ultraviolet light, and magnetic fields were applied to enhance enzyme activity. The most effective method was seen with UV light treatment (365 nm, 45 min) applied to rice bran peroxidase, achieving detoxification rates of 78.2% for AFB_1_ and 71.2% for AFM_1_ at 4 °C. This approach presents a promising, cost-effective strategy for aflatoxin mitigation in milk under refrigeration conditions, suitable for industrial applications.

A recent advance involves CotA laccase from *Bacillus licheniformis*, identified as an efficient AFM_1_ oxidase active directly in milk. Guo et al. [219] showed that CotA catalyzes C3-hydroxylation of AFM_1_ into the less toxic metabolites AFN_1_ and epi-AFN_1_. The enzyme achieved up to 86% oxidation in buffer and removed 83.5% and 65.1% of AFM_1_ in skim and whole milk, respectively, at 37 °C after 12 h.

Despite promising results in model systems, the efficacy of enzymatic reduction in milk remains limited due to acidic pH, enzyme instability, and protein–toxin interactions. Further optimization for use in food systems is needed for optimum reduction.

#### 7.2.4. Natural Additives

In addition to the use of probiotics and enzymes, natural feed additives are being investigated for their role as an alternative in reducing AFM_1_ levels in milk, especially due to their biodegradability and low toxicity. They act primarily through inhibiting AFB1-producing fungi in feed or through physical adsorption of AFM1 via polysaccharide- or chitin-based matrices [220].

Plant-based compounds, such as polyphenols, curcuminoids, and essential oils, have demonstrated varying reduction capacities mainly by inhibiting fungal growth and AFB1 biosynthesis. Girolami et al. [221] studied the effect of turmeric powder supplementation in Holstein-Friesian cows fed a low-AFB_1_ diet. Although the reduction was not significant, the addition of 20 g/day of turmeric powder lowered AFM_1_ levels by only 13% without affecting milk yield, composition, or somatic cell count. Beyond curcuminoids, essential oils represent another category of natural antimicrobials with strong inhibitory effects on aflatoxin-producing fungi. A blend of cinnamon, oregano, and lemongrass oils inhibited *Aspergillus flavus*, reducing AFB_1_ production by up to 73% in vitro, suggesting potential for practical application in grain storage [222]. Moreover, low concentrations of cinnamon essential oil (0.125%) reduced AFB_1_ by up to 90%, while also causing severe morphological damage to *Aspergillus flavus* hyphae and spores. Compared to other essential oils, such as clove, garlic, and peppermint, cinnamon was ten times more effective, suggesting its potential use as a natural, eco-friendly inhibitor of aflatoxin contamination [223]. However, essential oils are limited in dairy applications because of their strong aroma and potential sensory defects.

Exopolysaccharides (EPS) derived from probiotic fermentations also show promise as bio-adsorbents. Jiménez-Pérez et al. [224] evaluated EPS derived from probiotic fermentation of kefir grains, in binding AFM_1_ in milk and aqueous buffer. The addition of kefiran at a low concentration of 0.1 mg/mL resulted in an 81% reduction in AFM_1_ in milk after 6 h of interaction and 75% reduction in phosphate buffer. The result shows a high efficacy of EPS, likely due to their high surface area and polysaccharide composition. In addition, their food-grade status and prebiotic benefits make them suitable candidates for dairy formulations.

Marine-derived materials, such as chitin and shrimp shell, offer another class of natural binders. Marine polysaccharides remove AFM_1_ predominantly via adsorption through hydroxyl and amine functional groups, which interact with AFM_1_ via hydrogen bonding and electrostatic attraction [220]. Assaf et al. [198] examined these by-products in detoxifying AFM_1_ in UHT and PBS, and found that binding efficiency ranged from 14.29% to 94.74% depending on conditions, with lower temperatures improving binding efficiency, particularly for chitin, likely due to enhanced hydrogen bonding and stabilization of binding sites. A recent study has engineered chitin-derived hierarchical porous carbons with very high surface area and strong adsorption capacity for multiple mycotoxins, including AFB_1_ and B_2_. Thus, chitin can be valorized not only in its natural form but also as engineered porous carbons for effective aflatoxin adsorption. While these materials are biodegradable and low-toxicity, their use in milk remains limited by sediment formation [225].

Nanotechnology has also been used to enhance the effect of natural antimicrobials. In fact, nisin nanoparticles showed antifungal activity against *Aspergillus flavus* in Ras cheese compared to the use of pure nisin, achieving complete elimination by week 7 [226]. The primary mechanism is fungal suppression rather than direct AFM_1_ reduction, and therefore, it shows the potential of nanocarrier systems to target fungal contamination at its origin.

### 7.3. Physical Mitigation Techniques

Physical mitigation relies on processing and technological approaches applied directly to milk and dairy products to reduce AFM_1_ content. Unlike biological or chemical strategies, physical methods affect AFM_1_ distribution through protein partitioning, migration, moisture loss, etc. These techniques include irradiation, processing steps, barrier systems, and others to facilitate the toxin removal or alter its stability. Their feasibility largely depends on whether they preserve sensory quality, nutritional attributes, and structural integrity of dairy products. The following subsections outline how common processing steps and emerging techniques influence AFM_1_ levels (Supplementary Table S3).

#### 7.3.1. Processing Impact on AFM_1_

Dairy processing steps have an impact on AFM_1_, yet conventional methods often fail to eliminate the toxin, since it can be found in cheese or whey [227]. AFM_1_ is heat stable, and according to studies, it is unaffected by different processing techniques such as pasteurization, sterilization, and UHT treatment [32,35,216]. According to Harshitha et al. [228], high temperature treatments, like boiling and sterilization, only reduced AFM_1_ levels by up to 20%. In fact, complete degradation of AFM_1_ requires extreme temperatures between 237 and 306 °C, far beyond those used in food processing [19]. Even when processing temperatures are very high, reduction rarely exceeds 20 to 30% [229,230]. Therefore, dairy products retain AFM_1_ concentrations close to or above regulatory limits, especially if the raw milk is already contaminated with high levels of AFM_1_ [229]. This shows that such thermal techniques are insufficient in eliminating AFM_1_ and may require a combination with other mitigation techniques to achieve the desired reduction. Ewida et al. [114] studied several processing methods, such as carbonated water treatment, microwave heating, and lemon juice application. Microwave heating of aflatoxin-contaminated milk resulted in a modest reduction of 9.4%, while non-thermal methods, such as carbonated water treatment and lemon juice application, showed 54.9% and 43.9% reduction in aflatoxin in kareesh cheese. The acidic nature of citric acid enhanced aflatoxin degradation, making these approaches alternatives or complementary techniques to conventional processing.

The drying process involved in powdered milk production may lead to a concentration of AFM_1_ due to water removal [227]. A study in Morocco showed that all powdered milk samples were contaminated, with an average AFM_1_ level of 25.5 ± 12.06 ng/kg, a result higher than those in UHT milk (14.76 ± 10.21 ng/kg) [231]. These studies show that dehydration steps could contribute to the elevated AFM_1_ concentration in milk powder in addition to the initial contamination in raw milk.

During cheese production, AFM_1_ concentration increases significantly compared to the original milk used, due to the toxin’s strong affinity for casein, which causes it to be retained preferentially in the curd and results in significant concentration in the cheese product [232]. AFM1 interacts with casein micelles through hydrophobic interactions and hydrogen bonding, favoring its retention in the curd. The method of coagulation, whether using rennet, plant enzymes, or acids, affects AFM_1_ distribution because of its influence on casein micelle stability [229]. Furthermore, hard cheeses tend to concentrate AFM_1_ more than soft ones due to greater moisture loss. AFM_1_ concentration in hard cheese increases by 168% compared to the original milk, in comparison to an increase of 60.6% in soft cheese, during pressing and ripening [69,233]. In addition, cheeses produced from naturally contaminated milk show higher AFM_1_ than those made from artificially spiked milk, due to a stronger toxin–casein binding in natural contamination [233].

The use of starter cultures in cheese has contributed to the reduction in AFM_1_ levels [234]. Cheese produced without starter culture showed a 156.5% increase in AFM_1_, while those with starter culture showed only a 37% increase in AFM_1_, likely due to the detoxification and binding effect of lactic acid bacteria. However, results vary across studies. While some report higher AFM_1_ in cheese compared to milk, others found lower levels of AFM_1_ in curd and cheese [233]. Such inconsistencies affecting AFM_1_ retention in cheese are influenced by many factors, including moisture content, pH, renneting temperature, curd size, pressing time, and the presence of starter cultures [235]. Moreover, the presence of starter cultures can reduce the carry-over of AFM_1_ from milk into cheese, as LAB adsorb the toxin onto their cell walls and alter casein interactions [19,233]. For instance, reported AFM1 concentrations may vary depending on the analytical method used, with discrepancies often observed between ELISA and chromatographic techniques such as HPLC-FLD or TLC, especially in complex dairy matrices [233].

Ripening further affects AFM_1_. While some studies report AFM_1_ reduction during ripening, especially in cheeses produced with starter culture [19,233], others have observed stable or even increased AFM_1_ levels due to moisture loss and proteolytic activity [19,229,232]. These variations show that AFM_1_ behavior in cheese is highly dependent on production conditions, cheese type, ripening duration, contamination source, and the analytical methods employed.

On the contrary, a big portion of AFM_1_ can migrate to whey during cheese-making. In fact, up to 60% of AFM_1_ can transfer to whey during cheese-making, particularly when whey proteins retain their binding capacity, as observed in Cremoso Argentino cheese [236]. Einolghozati et al. [237] even reported a transfer of about 70.72% of AFM_1_ from raw milk into whey during cheese production, although this finding contrasts with many studies. In most cases, whey contains significantly less AFM_1_ (up to 49% lower), confirming that AFM_1_ binds more strongly to casein than to whey proteins [236]. These different results in the literature are probably due to differences in the manufacturing process, analytical techniques, curd particles in the whey, renneting temperature, pressing time, and form of milk contamination type, which may affect AFM_1_ levels during cheese production [238]. Given that whey is widely used in infant formula, protein powders, or beverages, understanding AFM_1_ distribution between curd and whey is very important for risk assessment.

Salting techniques, like brine, can enhance AFM_1_ migration from cheese to brine due to osmotic gradients and ionic strength effects, contributing to further toxin reduction in the curd. For example, Motawee & McMahon, ref. [239], observed up to 29% AFM_1_ reduction in Feta cheese over 60 days of brining, with enhanced effect under higher salt concentrations and ripening temperatures. Similarly, in traditional white cheeses such as Halloumi and Akkawi, Daou et al. [240] observed that brine storage (10%) contributed to a reduction in AFM_1_ levels in the curd. The authors suggested that this decrease may be attributed to ionic strength effects or dehydration-induced changes in protein interactions, which promote AFM_1_ migration from the curd into the liquid matrix. Storage also affects AFM_1_ dynamics. A study noticed that the AFM_1_ concentration in some types of cheese decreases during storage due to the toxin’s degradation over time [233], sometimes reaching 77.66%, likely due to microbial or enzymatic degradation [237]. This shows the potential of brining and storage strategies for natural detoxification of AFM_1_ in dairy products.

In fermented dairy products, reduction reached 80% in yogurt, attributed to a low pH, bacterial binding, and organic acid production by LAB [241]. El-Desouky and Kholif [214] reported that LAB fermentation of contaminated yogurt samples resulted in AFM_1_ reduction of up to 78.5% after five days of storage without affecting sensory properties. AFM_1_ reduction is affected by the type of strain, the product fermented, and the fermentation conditions.

In general, dairy products pose a higher AFM_1_ risk than liquid milk, due to toxin concentration during processing. Nonetheless, processing steps, such as the use of starter cultures, extended ripening, and brining, present techniques that can reduce AFM_1_ and should be considered as part of an integrated aflatoxin control strategy.

#### 7.3.2. Barrier System and Innovative Material

Other innovative physical mitigation techniques have emerged as promising approaches for reducing aflatoxin contamination in dairy products. Combining physical barrier materials with antifungal agents has shown potential in mitigating aflatoxins. Fayed et al. [242] explored the use of physical matrices (cellulose sheets) fortified with antimicrobial agents, such as Natamycin-loaded alginate nanoparticles to control the growth of *Aspergillus flavus* in cheese and aflatoxin B_1_ reduction. The mechanism relies on controlled antifungal diffusion from the polymer matrix and physical restriction of mold colonization, leading to a 78.6% reduction in total aflatoxins after 12 weeks. Nutritional and sensory properties of the cheese were largely preserved, suggesting good feasibility for ripened cheeses, although application is constrained to surface-ripened products. Other physical mitigation methods were studied, such as the use of DNA-conjugated magnetic beads to remove AFM_1_ from milk. In this system, AFM_1_ intercalates into DNA immobilized on magnetic beads, and the bead–toxin complexes are then recovered by magnetic separation. This approach achieved removal efficiencies of 95.5% in aqueous solution and 85.5% in milk at 10 µg/L. This technique is promising since it is non-toxic, efficient, reusable, and compatible with milk components [243].

#### 7.3.3. Irradiation-Based Technique

AFM_1_ levels in pasteurized milk were examined using radioactive granite (RG) and low-level gamma irradiation (LLGI). Radioactive granite (RG) can naturally emit low levels of gamma radiation, which can be used as a detoxification method for contaminated food matrices. In this approach, milk packs were placed inside a chamber surrounded by RG stones, allowing continuous low-level gamma irradiation (LLGI) without direct contact or chemical additives. The mechanism involves radiolytic cleavage of AFM_1_ functional groups, achieving a reduction of 51.5% after 4 days and up to 99% after 8 days of exposure, while maintaining the chemical and sensory quality of the milk. Although effective, the safety of degradation by-products requires further characterization, and translation to industrial continuous-flow systems remains uncertain [244]. Ultraviolet-C (UVC) light, with a wavelength range of 200–280 nm, is another physical method used for microbial inactivation and aflatoxin degradation by inducing photochemical reactions that break down their toxic structures. Nguyen et al. [245] reported that UVC treatment of skim milk at 254 nm reduced AFM_1_ concentrations by up to 50% after 20 min, achieving a safe level of (0.5 µg L^−1^). Furthermore, the use of UV-A LED technology (365 nm) demonstrated promising results in reducing both AFB_1_ and AFM_1_ in milk. Kurup et al. [246] treated contaminated milk with UVA and achieved a reduction of 78.2% for AFB_1_ and 65.7% for AFM_1_ under specific doses, without inducing cytotoxic effect on liver cells in vitro.

#### 7.3.4. Thermal and Non-Thermal Approaches

In addition to conventional heating, emerging thermal and non-thermal technologies are being explored for AFM_1_ reduction while preserving milk quality. One such method is thermoultrasound, which combines mild heat with ultrasound cavitation. Hernández-Falcón et al. [247] reported that thermoultrasound treatment at 20 kHz reduced aflatoxin M_1_ after 10 min of exposure by almost 99% in unhomogenized milk on day 1 while maintaining compliance with microbiological standards and preserving milk quality. A non-thermal technology, High Voltage Atmospheric Cold Plasma (HVACP) using dielectric barrier discharge, effectively reduces AFM_1_ by generating reactive species like ozone, hydroxyl radicals, and singlet oxygen that oxidatively degrade AFM_1_. Nikmaram and Keener [248] achieved over 87% AFM_1_ reduction in skim and whole milk after 3 min of treatment, followed by 4 h of storage. The technology’s result is enhanced by reactive species like ozone and hydroxyl radicals, which interact more with skim milk than with whole milk. Milk’s nutritional contents are preserved, but a slight pH reduction and lipid oxidation may occur, particularly in whole milk. Similarly, Nguyen et al. [245] demonstrated that HVACP effectively degrades AFM_1_ in skim milk, achieving up to 78.9% reduction with a modified atmosphere gas mixture in 20 min without altering milk color with reduced toxicity. High-pressure processing (HPP) has also been explored as a non-thermal strategy. Pressure-induced conformational changes in the matrix may promote partial destabilization or redistribution of the toxin, resulting in modest reductions. Pallarés et al. [249] reported modest reductions in aflatoxins (up to 24% for AFB_1_) in juice–milk beverages at 600 MPa. Although AFM_1_-specific evidence remains limited, HPP could play a complementary role in aflatoxin mitigation, especially when combined with other technologies.

Many techniques have shown positive results in aflatoxin mitigation in contaminated samples; however, issues related to safety, practicality, economic feasibility, and technological scalability must be addressed before adopting these techniques. Some techniques can have drawbacks, such as nutritional loss or ineffective removal of the toxin. Therefore, a combination of several techniques, targeting different points in the production process, appears to be the most promising pathway toward achieving effective aflatoxin control.

## 8. Conclusions

AFM_1_ remains a persistent challenge in dairy safety, especially in regions with high environmental risk factors and limited regulations. This review provided a comprehensive synthesis of global research trends, aflatoxin biological mechanisms, toxicity and regulation profiles, and mitigation strategies, supported by a bibliometric analysis of the last decade. The bibliometric findings revealed an increase in scientific interest, especially in countries experiencing high contamination levels, and highlighted the key contributors in driving the research forward. Nevertheless, reliance on Scopus data solely, potential keyword restrictions, and citation-based metrics introduce biases that may overlook regional studies and undervalue emerging research.

Despite a wide range of research on aflatoxin, complete elimination from the dairy chain remains unachievable. Mitigation strategies have shown benefits and drawbacks, and some of them require further validation in industrial settings. The prevalence studies confirm the global contamination by AFM1, with a striking difference between nations with strict regulations and those with an inadequate monitoring system. Emerging risks, such as combined contaminants and climate change leading to fungal growth, emphasize the urgency of a multifaceted approach to AFM1 management.

Future research in AFM_1_ management will depend on several factors. First, advances in rapid, cost-effective detection technologies, such as biosensors, nanotechnology-based tools, or AI-enabled monitoring platforms, will enhance AFM_1_ surveillance across the dairy supply chain. Second, unifying regulatory standards internationally will reduce disparities and ensure safer global trade. Third, sustainable mitigation strategies, such as probiotics, enzymatic detoxification, and valorization of agro-industrial by-products, offer promising eco-friendly alternatives. Moreover, predictive modeling of AFM_1_ occurrence, integrating climate variables, geospatial analysis, and meteorological data, must be utilized to anticipate contamination hotspots and adapt agricultural and dairy management practices proactively.

Notably, countries most vulnerable to climate change, especially in Sub-Saharan Africa and several arid MENA (Middle East and North Africa) states, remain underrepresented in AFM_1_ research. Screening and surveillance studies in these regions are needed to understand local exposure risks and to design context-specific mitigation strategies.

Finally, safeguarding the dairy sector against AFM_1_ will require a multifaceted approach that combines technological innovation, policy alignment, and international cooperation, ensuring both public health protection and long-term sustainability of dairy production worldwide.

## Figures and Tables

**Table 1:** Top 20 contributing countries in aflatoxin M_1_ dairy research.

Ranking	Country	Number of Publication	Percentage (% of 804)
1	Iran	137	17
2	China	89	11
3	Brazil	66	8.2
4	United States	54	6.7
5	Egypt	53	6.6
6	Pakistan	50	6.2
7	Italy	46	5.7
8	India	41	5
9	Turkey	41	5
10	Spain	36	4.5
11	Serbia	28	3.5
12	Ethiopia	21	2.6
13	Saudi Arabia	21	2.6
14	Germany	19	2.4
15	Kenya	19	2.4
16	Belgium	14	1.7
17	Greece	13	1.6
18	Mexico	13	1.6
19	Sweden	13	1.6
20	United Kingdom	13	1.6

**Table 2:** Leading affiliations by number of publications on aflatoxin M_1_.

Affiliation	Countries	Articles	Percentage of Articles out of 804
University of Sγo Paulo	Brazil	71	8.8
Islamic Azad University	Iran	69	8.6
Bahauddin Zakariya University	Pakistan	65	8.4
Institute of Animal Science	China	55	6.8
Kermanshah University of Medical Sciences	Iran	51	6.3
University of Novi sad	Serbia	44	5.5
Croatian Veterinary Institute	Croatia	42	5.2
Shahid Beheshti University of Medical Sciences	Iran	41	5.1
University of Veterinary and Animal Sciences	Pakistan	33	4.1
Nanchang University	China	32	4

**Table 3:** Most influential journals on aflatoxin research.

Ranking	Journal	Number of Publication	Impact Factor (IF) (2024)	Citescore (2024)
1	Food Control	76 (9.4%)	6.3	14.1
2	Toxins	55 (6.8%)	4.0	8.2
3	Food Chemistry	30 (3.7%)	9.8	18.3
4	Mycotoxin Research	20 (2.5%)	3.1	4.3
5	Journal of Dairy Science	17 (2.1%)	4.4	7.8
6	Food Additives and Contaminants: Part B—Surveillance	16 (2%)	2.5	5.2
7	International Journal of Dairy Technology	16 (2%)	2.8	5.5
8	World Mycotoxin Journal	11 (1.4%)	2.2	4.8
9	Journal of Food Composition and Analysis	10 (1.2%)	4.6	7.2
10	Journal of Food Safety	10 (1.2%)	1.8	4.2

**Table 4:** Top globally cited papers from 2015 through to 2025 according to the number of citations in Scopus (retrieved on August 2025).

Rank	Reference	Title	Year	Source	Cited by	Document Type
1	ALSHANNAQ et al. [45]	“Occurrence, Toxicity, and Analysis of Major Mycotoxins in Food”	2017	Environmental Research and Public Health	1019	Review
2	MARCHESE et al. [46]	“Aflatoxin B_1_ and M_1_: Biological Properties and Their Involvement in Cancer Development”	2018	Toxins	461	Review
3	EFSA Panel on Contaminants in the Food Chain (CONTAM) et al. [47]	“Risk Assessment of Aflatoxin in food”	2020	EFSA Journal	391	Article
4	HAQUE et al. [48]	“Mycotoxin contamination and control strategy in human, domestic animal and poultry: A review”	2020	Microbial Pathogenesis	323	Review
5	MAHATO et al. [21]	“Aflatoxin in Food and Feed: an Overview on Prevalence, Detection and Control Strategies”	2019	Frontiers in Microbiology	311	Review
6	ISMAIL et al. [49]	“Aflatoxin in foodstuffs: Occurrence and recent advances in decontamination”	2018	Food Research International	258	Review
7	FLORES-FLORES et al. [50]	“Presence of mycotoxins in animal milk: A review”	2015	Food Control	226	Review
8	IQBAL et al. [51]	“Aflatoxin M_1_ in milk and dairy products, occurrence and recent challenges: A review”	2015	Trends in Food Science and Technology	221	Review
9	CAMPAGNOLLO et al. [19]	“The occurrence and effect of unit operations for dairy products processing on the fate of aflatoxin M_1_: A review”	2016	Food Control	214	Review
10	BECKER-ALGERI et al. [52]	“Mycotoxins in Bovine Milk and Dairy Products: A Review”	2016	Journal of Food Science	168	Review

**Table 5:** Global occurrence of aflatoxin M_1_ in milk and dairy products.

Country	Type of Dairy Product	Positive Samples/Total Samples	Concentration of AFM_1_ (ng/L)	Samples Exceeding EU Limit (%)	Detection Method	Reference
Bangladesh	Raw milk	75/105 (71.4%)	5.0–198.7	23.8%	ELISA	Sumon et al. [122]
	Pasteurized milk	15/15 (100%)	17.2–187.7	73.3%		
	UHT milk	15/15 (100%)	12.2–146.9	73.3%		
	Yogurt	5/5 (100%)	8.3–41.1	0%		
	Milk powder	4/5 (80%)	5.9–7.0	0%		
Bangladesh	Raw milk	35/50 (70%)	22.79–1489.28	97.1%	ELISA	Tarannum et al. [115]
	Pasteurized milk	13/25 (52%)	18.11–672.18	46.1%		
	UHT milk	5/25 (20%)	25.07–48.95	0%		
Brazil	Cheese	28/28 (100%)	26–132	0%	HPLC-FLD	Silva et al. [123]
Brazil	Pasteurized and UHT milk	6/68 (8.8%)	15–227	1.4%	LC-MS/MS	Frey et al. [102]
Brazil	UHT	15/34 (44.11%)	150–550	100%	HPLC-FLD	Conteçotto et al. [124]
	Powdered milk	1/10 (10%)	1020	100%		
	Infant formula	1/16 (6.2%)	320	100%		
China	Pasteurized milk	1/294 (0.3%)	33.4	100%	Ultra-Performance Liquid Chromatography (UPLC) with C18 solid-phase FLD	Meng et al. [103]
	UHT	2/92 (2.2%)	38.7 36.5	100%		
	Infant formula	0/20 (0%)	Not detected (ND)	0%		
China	Pasteurized milk	40/93 (43%)	5–11.3	0%	ELISA	Xiong et al. [125]
	Extended shelf life milk	44/96 (45.8%)	5–16.5	0%		
	Donkey raw milk	0/70	-	0%		
Croatia	Raw milk	109/5817 (1.9%)	50.3–1100.0	100%	ELISA and UHPLC- FLD MS/MS	Bilandžić et al. [126]
Egypt	Raw milk	80/100 (80%)	BDL-105	7%	ELISA	Ibrahim et al. [127]
	Domiati Cheese	7/33 (21.2%)	BDL-99	18%		
	Karish cheese	33/33 (100%)	BDL-183	73%		
	Ras cheese	28/34 (82.3%)	BDL-250	50%		
Egypt	Raw milk	20/20 (100%)	2940–4560	100%	ELISA	Ewida et al. [114]
	Karish cheese	20/20 (100%)	3470–30,460	100%		
	Mish cheese	20/20 (100%)	3770–40,500	100%		
Egypt	Raw buffalo milk	43/56 (76.8%)	28–1200	39.29%	ELISA	Elsayed et al. [43]
France	Milk	All simulated milk batches assumed to contain AFM_1_	0.33–37.8	Less than 5%	-	Chhaya et al. [25]
India	Raw milk	204/300 (68%)	>500	100%	Charm ROSA Lateral Flow Test	Kumar et al. [60]
India	Raw milk	19/46 (41.3%)	ND-2913	84.2%	HPLC-FLD	Hattimare et al. [104]
	Pasteurized milk	6/15 (40%)	ND-1212	100%		
	UHT milk	18/52 (34.6%)	ND-1523	100%		
	Milk powder	2/10 (20%)	ND-2608	100%		
	Yogurt	3/10 (30%)	ND-303	100%		
Ghana	Raw cow milk	67/120 (55.8%)	60–3520	52.5%	HPLC-FLD	Kortei et al. [94]
Ghana	Wagashi (Traditional cheese)	11/18 (61.1%)	0.00–59.2 ± 2	5.56%	HPLC-FLD	Kortei et al. [128]
Ghana	Fresh cow milk	53/56 (94.6%)	61.8–1606.8	100%	HPLC-FLD	Nuhu et al. [105]
Greece (Thessaly)	Raw milk (cow, goat, sheep)	39/396 (10.1%)	7.94–105 (ELISA Kits) 7.96–75 (HPLC-FL)	Not stated	ELISA and HPLC-FLD	Malissiova et al. [107]
Greece	Infant/toddler milk	31/52 (59.6%)	2.03–9.38	0%	ELISA	Maggira et al. [129]
	Pasteurized milk	21/32 (65.6%)	2.04–17.84	0%		
	Feta cheese	7/25 (28%)	2.10–4.09	0%		
Hungary	Raw milk	191/278 (68.7%)	5–173	9.4%	ELISA	Buzás et al. [130]
	Processed milk	155/196 (79.1%)	5.3–100	0.5%	ELISA	
Iran (Tabriz)	Raw milk	8/8 (100%)	28.30–46.60	0%	HPLC-FLD	Behtarin & Movassaghghazani, [32]
	Pasteurized milk	8/8 (100%)	19.50–36.60	0%		
	UHT milk	8/8 (100%)	16.10–36.10	0%		
	Traditional yogurt	8/8 (100%)	35.30–50.20	25%		
	Pasteurized yogurt	8/8 (100%)	21.60–41.70	0%		
	Traditional cheese	8/8 (100%)	45.50–105.70	0%		
	Pasteurized cheese	8/8 (100%)	31.80–55.40	12.5%		
Iran (Ilam and Lorestan Provinces)	Raw Milk	40/40 (100%)	38.6–85.0	46.6%	HPLC-FLD	Aghebatbinyeganeh et al. [131]
	Pasteurized milk	40/40 (100%)	24.1–59.7			
	UHT milk	40/40 (100%)	21.4–69.4			
	Traditional cheese	40/40 (100%)	80.4–169.4	100%		
	Pasteurized cheese	40/40 (100%)	28.4–67.5	0%		
	Traditional Yogurt	40/40 (100%)	55.2–99.1	100%		
Iran (Tehran)	Powdered milk	24/25 (96%)	0.00–95.5	68%	HPLC-FLD	Movassaghghazani & Shabansalmani [91]
Iran (Golestan Province)	Camel milk	10/10 (100%)	57.10	57.5%	HPLC-FLD	Jorjani & Movassaghghazani [132]
	Raw milk	10/10 (100%)	72.81			
	Pasteurized milk	10/10 (100%)	34.73			
	UHT milk	10/10 (100%)	49.36			
Ireland	Milk	All simulated milk batches assumed to contain AFM1	0.00087–5.72	Around 1%		Chhaya et al. [25]
Italy (Sicily area)	Cow milk	0/180 (0%)	Below LOD	0%	HPLC-FLD	Messina et al. [13]
Italy (northern Italy)	Raw cow milk	At least 1057	<5 ng/L to >80 ng/L	0.7%	ELISA and HPLC-FLD	Ferrari et al. [133]
Italy	Cow milk	2244/3151 (71.2%)	9–146	0.9%	ELISA and HPLC-FLD	Roila et al. [119]
	Ewe milk	1424/5254 (27.1%)	6–239	1.1%		
	Cheesemaking cow’s milk	5817/8529 (68.2%)	6–208	2.2%		
Kenya	Raw milk	13/190 (6.84%)	>200 ng/L (None above 350 ng/L)	Not mentioned	Commercial lateral flow assay (LFA)	Smith et al. [134]
Kenya	Raw milk	Not stated/512	Mean value: Sub-Humid: 370.7 (n = 2), Humid: 52.9, Temperate: 34.6, Semi-Arid: 8.3	10%	ELISA	Sirma et al. [96]
Kenya	Raw milk, Pasteurized milk, UHT milk, Yogurt, Lala (fermented milk)	151/291 (51.9%)	<1.94–1068	50.2%	ELISA	Lindahl et al. [135]
Lebanon	Cow’s milk (Raw, Pasteurized, UHT)	422/722 (58.4%)	10.7–440.1	35.8%	ELISA, HPLC-FLD	Dominguez et al. [116]
Lebanon	Milk	Not specified	22.5–828.2	Not provided	HPLC-FLD, LC-MS, ELISA	Hoteit et al. [136]
	Yogurt	Not specified	-			
	Karicheh	Not specified	828.2 (max)			
	Labneh	Not specified	70 max			
Mexico	Raw milk	99/99 (100%)	10.6–73.8	11.3%	ELISA	Álvarez-Días et al. [137]
	Pasteurized milk	170/170 (100%)	10.6–73.8	10.3%		
Nigeria	Cow milk	23/23 (100%)	up to 81 ng/L	13%	LC-MS/MS	Akinyemi et al. [112]
	Goat milk	43/87 (49.42%)	up to 3108 ng/L	55%		
Pakistan	Raw milk	50/72 (69%)	344–741	100%	Lateral Flow Immunosensor	Ullah et al. [138]
Serbia	Cheese	42/60 (70%)	26–591	48.3%	ELISA, HPLC-FLD	Torović et al. [95]
Serbia	Raw milk	Not stated/385	~19.4–>242 ng/kg	46.2%	ELISA	Djekic et al. [139]
	Dairy product	7/556 (1.2%)		1.25%		
Spain	Natural yogurt	0/27 (0%)	0.007	0%	UHPLC-MS/MS technique	Rodríguez-Cañás et al. [140]
Spain	Milk (full-cream and raw)	0/191 (0%)	<25 (LOD)	0%	HPLC-MS/MS	Flores-Flores & González-Peñas [111]
Sudan	Cow milk	34/40 (85%)	35.3%: 48.5–97.1; 47.1%: 97.1–145.6	100%	ELISA	Yousof & El Zubeir, [113]
Turkey	Cheese	34/84 (40.5%)	251–559	100%	ELISA	Ergin et al. [141]

ELISA: Enzyme-Linked Immunosorbent Assay; HPLC-FLD: High-Performance Liquid Chromatography with Fluorescence Detection; UHPLC-MS/MS: Ultra-High Performance Liquid Chromatography–Tandem Mass Spectrometry; LC-MS/MS: Liquid Chromatography–Tandem Mass Spectrometry; LOD: Limit of detection; UHT: Ultra-High Temperature (milk); BDL: Below Detection Limit. *Note*: Although the EC has no specific ML for AFM1 in cheese, the milk limit of 50 ng/L was used as a reference for exceedance evaluation. For infant and toddler formula, a stricter EU maximum limit (ML) of 25 ng/kg (Regulation EC No 1881/2006) was applied.

**Table 6:** Comparative mitigation approaches for aflatoxin reduction in dairy systems.

	Mitigation Strategies	Reduction Range	Main Advantages	Main Limitations
**Chemical Mitigation**	Alkaline Agent (Ammoniation)	70–95% AFB_1_ reduction in feed	Highly effective Decrease AFM_1_ carry-over Low-toxicity by- products Suitable for large-scale feed treatment.	Not applicable to dairy products Requires controlled conditions Possible nutrient loss Ammonia handling and residue monitoring required.
	Oxidizing Agent (Ozonation)	10–55% AFM_1_ reduction in dairy. 50–99% AFB_1_ reduction in feed	Strong oxidizing degradation Improves microbial quality Environmentally friendly and residue-free	Moderate AFM_1_ reduction Quality changes Feasibility challenge at industrial scale Limited data on by-product safety in milk
	Oxidizing agent (Hydrogen peroxide H_2_O_2_)	40–100% depending on matrix and dose	Effective in milk and feed Cost-effective Strong oxidative degradation	Regulatory constraints Can oxidize milk nutrients Requires removal of residual H_2_O_2_
	Adsorbents (Bentonite)	40–90% reduction in AFM1 carry-over (via AFB_1_ binding)	Physical adsorption No toxic by-products Safe and regulatory-approved in feed Low cost	Efficacy varies with mineral composition and particle size Not used in milk processing
	Other Adsorbents (Zeolite, Composite, Mineral–Organic Blends)	15–60% AFM_1_ reduction/carry-over reduction	Multimodal adsorption mechanisms Safe, inert mineral bases Can enhance binding via MOS, yeast cell walls, or polysaccharides	Efficacy varies with formulation and contamination level Potential micronutrient interactions Reduced performance at high toxin loads Not used for milk
**Biological Mitigation**	LAB lactic acid bacteria	20–100% AFM_1_ reduction	Safe Residue-free Effective even with heat-treated/non-viable cells Potential synergistic effects	Binding is reversible Strain-specific performance Limited industrial-scale validation
	LAB-yeast Synergies)	70–100% AFM_1_ reduction	Faster and more complete removal than yeast or LAB alone Broad range of adsorption sites Safe	Adsorption is reversible Matrix dependence, High variability among strain combinations Limited industrial-scale validation
	Bifidobacteria and Cell-Wall Components	20–96% AFM_1_ reduction	Safe Cell-wall fractions (peptidoglycan) may outperform live cells Suitable for products where microbial growth is undesirable	Strain- and matrix-dependence Adsorption is reversible Limited industrial-scale validation
	Synergistic strategies (Probiotics + Nanoparticles/Surfactants)	15–100% reduction	Enhanced adsorption capacity compared to microbes alone Potential stabilization of binding	Mostly in vitro Safety and regulatory acceptance of nanoparticles/surfactants remain unclear Adsorption can be reversible Industrial scalability not yet established
	Indirect Biological Mitigation via Feed (AFB_1_-degrading microbes)	83–90% AFB_1_ degradation in feed (20–30%) AFM_1_ reduction	Prevents AFM_1_ formation at the source (feed) Does not affect milk composition or yield Practical for on-farm application	Effectiveness depends on AFB_1_ level Degradation by-products require safety evaluation Variable results across feeding systems
	Enzymes and Bioactive Compounds	Up to 83.5% AFM_1_ reduction	Chemical degradation of AFM_1_ Does not alter milk composition or yield Reduces AFB_1_ bioavailability before milk synthesis	Enzyme activity depends on pH, temperature, presence of inhibitors or metal ions, making industrial use challenging. Degradation by-products must be identified and proved safe
	Natural Additives (plant compounds, essential oils, EPS, chitin-based adsorbents)	AFM_1_ in milk: 10–80% AFB_1_ inhibition in feed up to 70–90%	Natural and biodegradable Strong antifungal activity reducing AFB_1_ formation Some materials (EPS, chitin-based adsorbents) show good AFM_1_ binding.	Highly variable efficacy Limited direct action on AFM_1_ for many plant compounds; Sensory issues with essential oils
**Physical Mitigation**	Processing impact	0–80% depending on the process	Brining and ripening can naturally lower AFM1 No chemical residues	AFM1 is heat-stable, Drying concentrates AFM1 Effects vary widely by cheese type, pH, moisture, strain, and manufacturing conditions
	Barrier systems (natamycin-loaded alginate nanoparticles on cheese surface)	Around 78.6% total AF reduction	Strong antifungal protection Suitable for ripened cheese	Only surface active Require long ripening time
	Barrier systems (DNA-conjugated magnetic bead adsorption	95.5% (water); 85.5% (milk at 10 µg/L)	Non-toxic, reusable, Effective in complex milk matrices	Currently at experimental stage Higher cost and technical complexity
	Irradiation-Based Techniques	50–99%	No chemical residues, Non-thermal treatments Effective reduction	Incomplete AFM1 degradation Potential sensory changes Unclear toxicological profile
	Thermal and Non-Thermal Approaches	Up to 99%	High AFM1 reduction potential under optimized conditions Preserve nutritional and sensory quality (non-thermal methods) Fast processing with no chemical residues Compatible with existing dairy technologies	Reduction efficiency varies with milk composition and processing conditions Incomplete removal for some methods (HPP) Possible pH or oxidation changes High equipment cost and limited industrial validation

Reduction ranges reflect laboratory and pilot-scale studies; industrial-scale validation remains limited for several emerging technologies.

## Data Availability

The raw data supporting the conclusions of this article will be made available by the authors on request.

## Supplementary Materials

The following supporting information can be downloaded at https://www.mdpi.com/article/10.3390/foods15010166/s1, Table S1: Chemical control methods for AFM_1_; Table S2: Biological control methods for AFM_1_; Table S3: Physical control methods for AFM_1_.

## Abbreviations

The following abbreviations are used in this manuscript:

AFAR	Aflatoxin aldehyde reductase
AFB_1_	Aflatoxin B_1_
AFL	Aflatoxicol
AFM_1_	Aflatoxin M_1_
BDL	Below detection limit
BDP	Biodegradation product
BENT	Bentonite
CBENT/CA	C combination with bentonite and activated carbon
CFU	Colony forming unit
CYP450	Cytochrome P450 enzymes
DOI	digital object identifiers
EFSA	European Food Safety Authority
ELISA	Enzyme-Linked Immunosorbent Assay
EPS	Exopolysaccharides
FAO	Food and Agriculture Organization
FDA	Food and Drug Administration
FLD	Fluorescence detection
FUM	Fumonisin
GRAS	Generally recognized as safe
GSTs	Glutathione S transferases
HBV	Hepatitis B virus
HCC	hepatocellular carcinoma
HPLC	High-Performance Liquid Chromatography
HPP	High-pressure processing
HSCAS	hydrated sodium calcium aluminosilicate
HVACP	High Voltage Atmospheric Cold Plasma
ICSE	Inorganic Composite Sorbent Extractant
IARC	International Agency for Research on Cancer
IF	Impact factor
IGF	Insulin-like growth factor
JECFA	The Joint Committee between FAO and WHO Experts on Food Additives
RH	Relative humidity
ROS	Reactive oxygen species
LAB	Lactic acid bacteria
LC-MS/MS	Liquid Chromatography–Tandem Mass Spectrometry
LD	Linear dichroism
LFA	Commercial lateral flow assay
LLGI	low-level gamma irradiation
LOD	Limit of detection
LOQ	Limit of quantification
MBNC	Magnetic bentonite nanocomposites
MB	Modified bentonite
MBNC	Magnetic bentonite nano-composite
MC-LR	Microcystin-LR
mEH	Microsomal epoxide hydroxylase
MENA	Middle East and North Africa
ML	Maximum limit
MOE	Margin of exposure
MOS	Mannan oligosaccharides
Mt-CS/CFS NSs	Metformin–chitosan/silica-cobalt ferrite nanospheres
NADPH	Nicotinamide adenine dinucleotide phosphate
NSFC	National Natural Science Foundation of China
MX	Multi-toxin binder mixes
NB	Natural bentonite
ND	Not determined
NP	Nanoparticles
OTA	Ochratoxin A
PBS	Phosphate-buffered saline washes
PEF	Pulsed electric fields
PG	Peptidoglycan
PTCC	Persian-type culture collection
RG	Radioactive granite
RSM	Reconstituted skim milk
rPODs	Recombinant peroxidases
SM	Sorbitan monostearate
SODs	Superoxide dismutases
UHT	Ultra-high temperature
UPLC	Ultra-Performance Liquid Chromatography
UV	Ultraviolet
USDA	United States Department of Agriculture
WHO	World Health Organization
YCW	Yeast cell wall
ZEN	Zearalenone
