# Supplementary material for: Mapping Global Research Trends on Aflatoxin M1 in Dairy Products: An Integrative Review of Prevalence, Toxicology, and Control Approaches
Source: Foods. 2026 Jan 3;15(1):166. doi: 10.3390/foods15010166 (PMC12785589; doi:10.3390/foods15010166)
Supplement: Supplementary file 1 [file foods-15-00166-s001.zip › foods-4023816-supplementary.pdf]

Supplementary table 1. Chemical Control Methods for AFM_1

Mitigation Strategy	Dairy Matrix	Strengths	Limitations	Reduction Level	Reference
ICSE (Inorganic Composite Sorbent Extractant) + Ammonia(4% $NH_3 \cdot H_2O$)	Animal Feed (Peanut cake)	High AFB_1 reduction; Nutrient retention	Slight cytotoxicity from ammonia residue	91.48% AFB_1 reduction	Xie et al. [1]
Ammoniation	**Animal feed (grain mix**: corn + rice + barley)	High AFB_1 reduction	Requires specialized equipment, potential protein loss, regulatory approval needed	91.2% AFB_1 reduction	Zhang el al. [2]
Ammoniation	**Animal feed (Groundnut Press cake)**	High AFB_1 reduction	Effect on nutritional quality unclear	Up to 96.3% AFB_1	Schrenk et al., [3]
Ozonation	Aqueous model system	Rapid AFB_1 degradation; no toxic residues	Tested in model systems, not in dairy matrices	Complete degradation of AFB_1 in 3-7 min at 13.5 ppm O_3	Agriopoulou et al.[4]
Ozonation	Milk spiked with AFM_1 (0.56 µg/kg)	AFM_1 reduction and reduced microbial count	Decreases β-carotene content, visible color change	Up to 50% AFM_1 (5 min, 80 mg/min O_3)	Mohammadi et al. [5]
Ozonation	Milk and whey concentrate	AFM_1 reduction, microbial load reduction	Incomplete degradation of AFM_1, possible texture/physicochemical changes	18.9% AFM_1 reduction	Sert & Mercan, [6]
Ozonation + UV + PEF (Ultraviolet + Pulsed Electric Fields)	Acidophilus milk	High AFM_1 reduction ; Minimal impact on milk nutrients	Effectiveness depends on optimization	up to 96.1% AFM_1 reduction	Khoori et al. [7]

Hydrogen Peroxide	Raw and naturally contaminated milk	Potential complete AFM_1 degradation under optimal conditions	High doses exceed legal limits; potential loss of nutrients and altered organoleptic properties	0-100% depending on H_2O_2, additives, and temperature	Marth & Applebaum, [8]
Hydrogen Peroxide + UV	Milk contaminated with AFM_1	High AFM_1 reduction with combined treatment; low residue	Overuse can impact sensory/nutritional quality	Up to 89% AFM_1 reduction (0.05 g/dL H_2O_2 + 20-min UV)	Shen & Singh, [9]
Bentonite supplementation	Animal feed	Improved nutrient digestibility, increased milk quality, reduced AFM_1 in milk	No significant effect on milk yield; less effective than montmorillonite	Up to 64.5% reduction in AFM_1	Gouda et al., [10]
Montmorillonite supplementation	Animal feed	Higher digestibility, best AFM_1 reduction, improved ruminal fermentation and blood glucose	Requires regular supplementation; slightly increased fecal AFB_1 excretion	Up to 82.2% reduction in AFM_1 in milk	Gouda et al., [10]
Bentonite (di- and tri-octahedral smectites)	Animal feed	Safe at 20,000 mg/kg in feed, high AFB_1 binding in vitro; approved for use in EU	No adequate in vivo efficacy data	90-95.3% **binding** of AFB_1 in vitro (pH 5)	Rychen et al., [11]
Bentonite	Animal feed	Highest reduction in AFM_1 transfer and milk concentration	Variable binder:AFB_1 ratio; excretion not always reduced	39-40% AFM_1 reduction in milk	Kihal et al. [12]
Hydrated sodium calcium aluminosilicate	Animal feed	Decreases AFM_1 indices	potential nutritional interactions	35% AFM_1 reduction	

Yeast cell wall (YCW)	Animal feed	some reduction in AFM_1; lowest excretion	Lowest efficacy; possibly reversible binding at high pH	25% AFM_1 reduction	
Mixed binders (MX)	Animal feed	Synergistic effects; moderate reduction in AFM_1 indices	No standard formulation; high variability	30-35% AFM_1 reduction	
Toxin binder (Exal® + bentonite, 50 mg/day)	Animal feed	AFM_1 reduction in milk; improve serum IgG response	Minor changes in biochemical markers	No percentage reported	Wakad et al. [13]
Processed bentonite (G.Bind™)	Animal feed	AFM_1 reduction in milk and transfer rate over 1-2 weeks	Short study duration	AFM_1 reduced from 153 to 47 ppt; transfer rate reduced from 1.17% to 0.39% (2 weeks)	Soufiani et al. [14]
Size-fractionated raw bentonite (<5 µm)	Animal feed	Similar to commercial adsorbent; reduced AFM_1 carry-over to 0.43%	Study tested at low AFB_1 levels (41 µg/kg DM); short duration (14 days)	AFM_1 excretion reduced to from 0.52% to 0.43%, in AFB_1-only group	Hajmohammadi et al. [15]
L. paracasei + bentonite (CBENT) or *B. coagulans* + bentonite (CBENT)	Milk (spiked)	Up to 100% AFM_1 removal; stable complexes; effective at 109 cfu/ml	Not evaluated for sensory effect	Up to 100% AFM_1 removal	Muaz & Riaz, [16]
Acid/heat-killed LAB + Activated Carbon (AC)	Milk	Very high AFM_1 reduction with L. cremoris + 0.1% AC; minimal milk	Requires combined application; strains specific	Up to 97.6% AFM_1 reduction	Muaz et al. [17]

		composition change			
Activated Carbon (AC)	Milk	High AFM_1 reduction; negligible effect on milk composition	Lower efficacy than combined treatment (LAB+AC)	78.2% AFM_1 reduction	
Bentonite (BENT)	Milk	Easy to apply	Lower efficacy than combined treatment	35.8 % AFM_1 reduction	
NovaSil® (Calcium montmorillonite)	Animal feed	Field-proven; High AFM_1 reduction; improves perceived milk safety	Efficacy depends on dose and contamination level	Around 90% AFM_1 reduction	Anyango et al. [18]
NovaSil Plus® (Calcium montmorillonite clay)	Animal feed	Dose-dependent AFM_1 reduction; no impact on milk yield/ composition/ vitamins	Requires monitoring and high doses efficacy	55%-68% AFM_1 reduction	Maki et al. [19]
Bentonite (aluminosilicate clay, 30 g/day)	Animal feed	AFM_1 reduction in milk; Improves milk yield (+17.12%); Enhances immune markers	Potential relative dehydration	66.7% AFM_1 reduction	Oliveira et al. [20]
Montmorillonite-rich clay product (containing vermiculite, nontronite, and montmorillonite	Animal feed	AFM_1 reduction in milk, feces, and rumen fluid; improved liver enzyme markers	Did not maintain milk yield or feed efficiency at higher clay levels	19-42% AFM_1 reduction; AFM1 transfer reduced from 1.37% to 0.74%	Sulzberger et al. [21]
Natural Bentonite (NB)	Animal feed	Improved yield and milk components (fat, lactose);	No effect on plasma metabolites;	AFM_1 carryover reduced	Ibrahimi Khoram Abadi &

		slight AFM_1 carryover reduction	slight reduction only	from 0.67% to 0.65%	Heydari, [22]
Modified Bentonite (MB)	Animal feed	Further improves milk quality	Still less effective than MBNC	AFM_1 carryover reduced from 0.67% to 0.64%	
Magnetic Bentonite Nanocomposite (MBNC)	Animal feed	Best milk yield and composition; strongest AFM_1 reduction; no health issues	Requires synthesis and processing of nanomaterials	AFM_1 carryover reduced from 0.67% to 0.60%	
Kaolin Clay	Raw milk (spiked)	High AFM_1 reduction; minimal nutrients change; safe and natural	Slight decrease in milk fat and protein at higher doses; less effective than bentonite at same doses	86.12% – 93.28% AFM_1 reduction	Moussa et al. [23]
Calcium Bentonite Clay	Raw milk (spiked)	Higher removal efficacy than kaolin; milk maintain composition	Slight non-significant decrease in fat content	93.69% - 97.71% AFM_1 reduction	
Bentonite sample 1 HAFR 3	Raw milk (spiked)	High adsorption efficacy; effective within 12 h; minimal impact on milk	slight changes in milk composition	Up to 98.5% AFM_1 reduction	Hamad et al. [24]
Bentonite Sample 2 HAFR 1	Raw milk (spiked)	High efficacy (95%); lower cytotoxicity	Slightly less effective than HAFR 3	Up to 95% AFM_1 reduction	
Clay Bentonite	Milk	High AFM_1 and OTA removal	slight impact on milk protein	68% AFM_1 reduction	Abdelnaby et al. [25]
Roasted Date Pit	Milk	Good removal of AFM_1 and OTA	Less effective than bentonite; limited data in milk	56% AFM_1 reduction	

Chitosan nanoparticles	Milk	Minimal impact on milk composition	Lowest efficacy among the tested adsorbents	12% AFM_1 reduction	
Mycosorb® (glucomannan, yeast cell wall)	Animal feed	Significant reduction in AFM_1 secretion	Tested only on Nili-Ravi buffaloes	47% reduction in AFM_1 secretion	Naveed et al. [26]
Fixar® Viva (Silicates and yeast cell wall component)	Animal Feed	Moderate AFM_1 reduction	No improvement in milk production reported	39% reduction in AFM_1 secretion	
T5X® (Clay, antioxidant, yeast extract, vitamins, betaine, minerals)	Animal Feed	Lower AFM_1 reduction	Least effective among tested binders	35% reduction in AFM_1 secretion	
Mycofix®: blend of bentonite (DOM), enzymatic detoxifiers, yeast (T. mycotoxinivorans), seaweed extract, and silymarin	Animal feed	Reduce AFM_1 concentration in milk and AFM_1 total excretion	Did not fully prevent carry-over; high AFM_1 excretion persist	Decrease AFM_1 from 98.3 to 76.5 µg/day; reduce carry-over rate from 4.60% to 3.44%	Aslam et al. [27]
Solis Mos® (SM) - sodium montmorillonite + yeast + MOS + vitamin E	Animal feed	reduced AFM_1 transfer at low AFB_1 dose; no effect on milk yield/composition	Ineffective at higher AFB_1 dose (40 µg/kg); effect is dose-dependent	At 20 µg/kg AFB_1: reduce AFM_1 by 16%, excretion by 18.3%, and transfer by 17.9%	Xiong et al. [28]
Clinoptilolite (Zeolite)	Animal feed	effective in field conditions; smaller particle size enhances binding	lower reduction compared to some bentonites; optimal particle size needed	Mean 56.2% AFM1 reduction in	Katsoulos et al. [29]
Montmorillonite + diatomite	Animal feed	Reduced AFM_1 concentration,	Tested at 8 µg/kg AFB1 only;	50.5% AFM_1	Cha et al. [30]

		excretion, and transfer rate; no negative effect on milk yield, composition	efficacy at higher levels unknown	reduction; decrease transfer rate from 1.16% to 0.57%	
Montmorillonite + diatomite + yeast cell wall + sodium alginate)	Animal feed	Reduced AFM_1 in milk, no effect on cow performance or health	Still exceeded EU MRL in some milk samples; limited data across AFB_1 levels	45.2% AFM_1 reduction in milk andtransfer rate from 1.16% to 0.63%	
Mycotoxin Sequestering Agent (Antitox CooPil®)	Animal feed	Reduction in AFM_1 levels and carry-over rate; no adverse effect on milk production, composition, or cow health	Effective only under low natural contamination	50% AFM_1 reduction; carry-over rate reduced by 65%	Costamagna et al. [31]
Mt-CS/CFS Nanospheres (Montmorillonite–chitosan nanospheres enriched with cobalt ferrite–silica)	Milk and aqueous solution	High adsorption capacity (Q_{max} = 3.891 mg/g), 91.1% removal of AFM_1 in milk, preserves milk quality, reusable, effective over wide pH range (3–9)	Requires magnetic separation; synthesis is multi-step and may not be industrially scalable	91.1% AFM_1 reduction	Moradian et al. [32]

ICSE: Instant Catapult Steam Explosion.

Supplementary table 2. Biological Control Methods for AFM_1

Mitigation Strategy	Dairy Matrix	Strength	Limitations	Reduction Level	Reference
S. boulardii	Reconstituted skim milk	High AFM_1 reduction	Temperature and strain dependent	Up to 96.88% AFM_1 reduction and 100% in combinations	Rezasoltani et al. [33]
L. acidophilus	Reconstituted skim milk	Effective even at chilled conditions	Lower efficacy than yeasts; strain-dependent	Up to 71.46% AFM_1 reduction	
L. casei	Reconstituted skim milk	Significant removal at higher toxin levels and temperatures	Lower efficacy at lower temps and shorter durations	Up to 64.31% AFM_1 reduction	
L. plantarum, L. brevis, and mixture (viable)	Raw milk	Indigenous strains; moderate reduction	Reduction requires long contact time (up to 72 h)	35-49% AFM_1 reduction	Erfanpoor et al. [34]
Postbiotics, parabiotics, and encapsulated *L. paracasei* KC39	Reconstituted milk powder	Strong antifungal and detox effects	Data mainly in vitro	AFM_1: 25-50%	Fahim et al. [35]
W. confusa H1 (viable and heat-treated)	reconstituted skim milk	High AFM_1 binding; EPS-producing strain	Mainly qualitative milk data	78% AFM_1 reduction	Chaudhary & Patel, [36]
L. plantarum S2 (viable and heat-treated)	reconstituted skim milk	Good AFM1 binding at warm and cold conditions	Slightly lower reduction at cold temperature	72% AFM_1 reduction	
Lactobacillus rhamnosus	reconstituted skim milk	High binding capacity	Binding may be reversible	Up to 91.82% AFM_1 reduction	Khadivi et al.[37]

Lactobacillus plantarum	reconstituted skim milk	Strong binding at lower concentration	Lower binding at higher temperature and concentration	Up to 89.33% AFM_1 reduction	
Saccharomyces boulardii	reconstituted skim milk	Highest single-strain binding	Slight drop in efficacy at 109 CFU/mL	Up to 93.2% AFM_1 reduction	
L. rhamnosus + *L. plantarum*	reconstituted skim milk	Strong Synergistic binding	Binding affinity ratio- and time-dependent	Up to 95.86% AFM_1 reduction	
L. rhamnosus + *L. plantarum* + *S. boulardii*	reconstituted skim milk	Achieved complete AFM_1 removal	Reduction declined from 100% to 91.7% with longer exposure	Up to 100% AFM_1 reduction	
Heat-killed LAB (*L. rhamnosus* + *L. lactis)*	Minas Frescal cheese	High AFM_1 reduction	Slightly less effective than yeast	94% AFM_1 reduction	Gonçalves et al. [38]
Heat-killed *Saccharomyces cerevisiae*	Minas Frescal cheese	Achieved 100% AFM_1 reduction over storage (day 20)	Slower action	100% AFM_1 reduction	
Heat-killed LAB + *S. cerevisiae*	Minas Frescal cheese	Achieved 100% AFM_1 reduction by day 10	Same final reduction as yeast alone, no synergistic effect	100% AFM_1 reduction (Day 10)	
S. thermophilus and *L. delbrueckii subsp. bulgaricus*	Milk	Very effective during fermentation	Ineffective in already fermented yoghurt	90–100% AFM_1 reduction	Seyedjafarri, [39]
Lactobacillus plantarum (C1 strain)	In vitro assay	High probiotic potential; antimicrobial properties	In vitro only	43.2% AFB_1 reduction	Zamani et al. [40]
Chitosan nanoparticles (2.5 mg/mL)	In vitro assay	effective at pH 7; biodegradable; non-toxic	Less effective alone	Around 22% AFB_1 reduction	

L. plantarum C1 + nanochitosan (synergistic combination)	In vitro assay (AFB_1-contaminated medium)	Strong synergy	In vitro only	Up to 69% AFB_1 reduction	
Bacillus subtilis YGT1	In vitro (LB broth)	High degradation of AFB_1	Not tested in real dairy or feed matrices	83.8% AFB_1 reduction	Al-Mamari et al. [41]
Bacillus subtilis ANSB060 biodegradation product (BDP)	contaminated cow feed with AFB_1	Reduces AFM_1 excretion	Short-term study	27–28% AFM_1 reduction	Guo et al. [42]

Supplementary table 3. Physical Control Methods for AFM_1

Mitigation Strategy	Dairy Matrix	Strengths	Limitations	Reduction Level	Reference
Skimming and Pasteurization	Naturally contaminated milk	Common Practice, preserve milk safety	No significant effect on AFM_1	0%	Harshitha et al. [43]
Boiling & sterilization	Naturally contaminated milk	High temperature treatments	Limited reduction	Up to 20%	
Fermentation (*Streptococcus thermophilus*)	Naturally contaminated milk	-	Minor reduction	Up to 8%	
Pasteurization	Milk	Widely used	No reduction	0%	Massarolo et al. [44]
Cheese-making + Ripening (Camembert)	Camembert cheese	Simulates realistic cheese processing	AFM_1 concentrated during ripening	Increased from <LOQ to 9.9 ng/g	
Cheese-making + Ripening (Morbier)	Morbier cheese	Ripening with LAB	Depends on starter culture strain and ripening time	Decrease by ~40% (2.64 to 1.46 ng/g)	
Pasteurization	milk	-	Did not significantly reduce AFM_1 levels	No significant reduction	Zebib et al. [45]
Traditional fermentation and boiling (cottage cheese production – Ayib)	Cottage cheese	Coagulation and pH drop reduce AFM_1	AFM_1 still detected in 25% of samples above EU limit	AFM_1 levels significantly lower than milk	

Microwave heating	Contaminated milk	accessible	Modest reduction	9.4% total aflatoxin reduction	Ewida et al. [46]
Carbonated water	Kareish cheese	Significant AF reduction; first novel trial	water composition and contact time dependent	54.9% total aflatoxin reduction	
Lemon juice addition	Mish cheese	accessible	Lesser efficacy than carbonated water;	43.9% total aflatoxin reduction	
Mozzarella, Asiago d'Allevo , Taleggio, caciotta processing	Milk	Lower AFM_1 concentration factor	AFM_1 remain stable	No reduction	Stella et al. [47]
Soft cheese processing	Naturally contaminated raw milk	-	AFM_1 concentration in cheese 5.5× higher than milk	No reduction. 44.6% remained in cheese, 55.4% in whey.	Costamagna et al. [48]
Lactic cheese Processing	Naturally contaminated raw milk	Large AFM_1 loss during production and storage	High AFM_1 transfer to whey (70.72%)	93.58% of total AFM1 removed from milk; 6.42% in cheese	Einolghozati et al. [49]
Brine Storage of lactic cheese	cheese	Further AFM_1 reduction over storage	Brine composition and time-dependent behavior may vary	77.66% reduction in AFM_1 during storage	
Yogurt processing	Yogurt	Significant reduction in AFM_1 during fermentation	Reduction plateaus after day 7; not all AFM_1 removed	AFM_1 reduction ranged from 44.5% to 55.6% after 14 days	Tahoun et al. [50]

Brining	Feta cheese	Enhances AFM_1 migration to brine	Requires long ripening time (up to 60 days); partial reduction only	Up to 29% AFM_1 reduction after 60 days	Motawee & McMahon [51]
Packaging with Natamycin-loaded alginate nanoparticles	Surface of Egyptian Romy cheese during 12-week ripening	Complete fungal growth inhibition after 8 weeks; preserved sensory qualities	Primarily effective at surface level; long ripening period	78.6% reduction in total aflatoxin	Fayed et al. [52]
DNA-conjugated magnetic bead	In aqueous solution and contaminated milk	High removal efficiency	Application still at experimental scale	95.5% in water, 85.5% in milk	Huang et al. [53]
Low-level gamma irradiation (LLGI) via radioactive granite (RG)	Pasteurized milk	High AFM_1 reduction without affecting milk's sensory or chemical quality	Requires 4–8 days of exposure; slower than other treatments	51.5% reduction after 4 days; 99% reduction after 8 days	Hassanpour et al. [54]
UVC (254 nm) treatment	Skim milk	Effective reduction of AFM_1; No milk pH change	Color of milk is affected	50–70% after 20 minutes	Nguyen et al. [55]
UV-A LED irradiation (365 nm)	Whole milk spiked with AFM_1 and AFB_1	quantifiable dose-response; no toxic by-products detected	Incomplete degradation (~65% for AFM_1); high UV dose required; sensory impact not fully assessed	65.7% for AFM_1 at 857 mJ/cm^2	Kurup et al. [56]

Thermoultrasound	Whole milk	Preserves physicochemical and microbiological quality; significant AFM_1 decrease	Effectiveness varies with treatment time and homogenization	Up to 62% aflatoxin reduction	Hernández-Falcón et al. [57]
High Voltage Atmospheric Cold Plasma (HVACP)	Skim milk	Reduces AFM_1 significantly; minimal nutrient change	Slight decrease in milk pH; Requires high-voltage equipment	Up to 78.9% with MA65 gas in 20 min treatment	Nguyen et al. [58]
HVACP	Skim and whole milk	Rapid AFM_1 degradation with short treatment (3–5 min); Minimal impact on nutrients	Slight color and pH changes with prolonged storage	Up to 92.8% in skim milk 87.8% in whole milk	Nikmaram & Keener, [59]

AFM1: aflatoxin M1; AF: aflatoxin; LOQ: limit of quantification; UVC: ultraviolet C; HVACP: high-voltage atmospheric cold plasma
